# Supplementary material for: Blood pressure trends following birth in infants born under 25 weeks’ gestational age: a retrospective cohort study
Source: BMJ Paediatr Open. 2024 Mar 25;8(1):e002438. doi: 10.1136/bmjpo-2023-002438 (PMC10966797; doi:10.1136/bmjpo-2023-002438)
Supplement: Supplementary data [file bmjpo-2023-002438supp001.pdf]

Blood pressure trends following birth in infants born under 25 weeks' gestational age - Supplementary Material

Emma Persad, MD, Björn Brindefalk, PhD, Alexander Rakow, MD, PhD

Figures

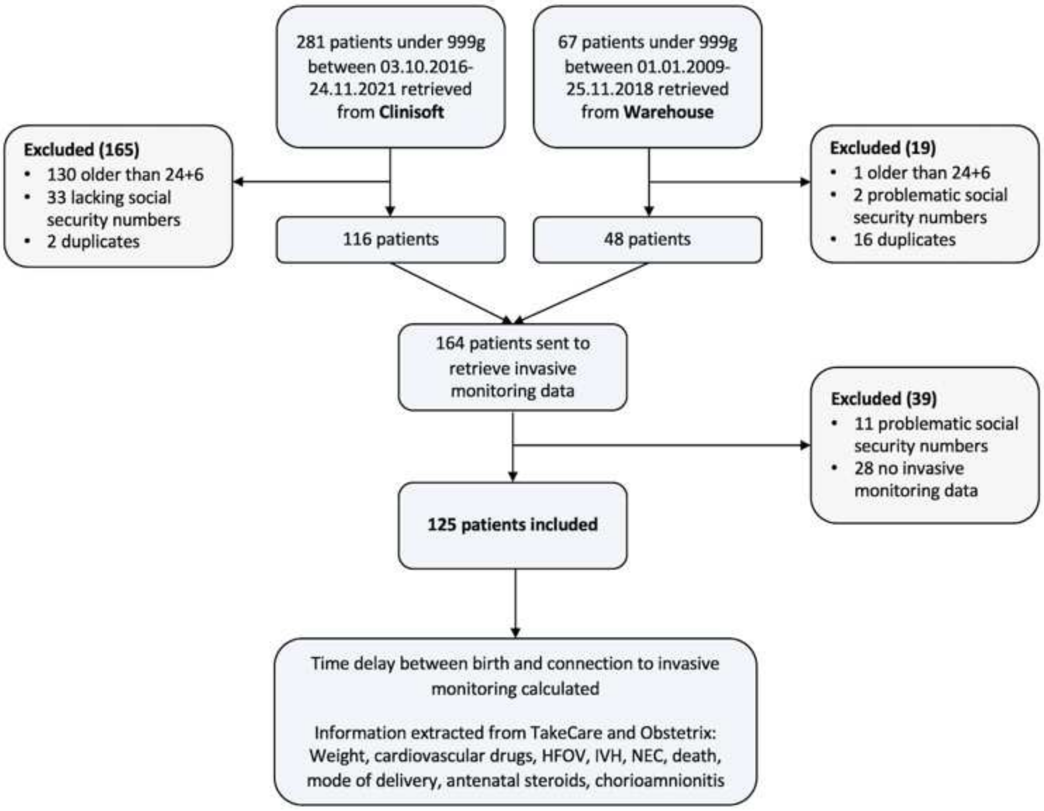

Supplementary Figure 1. Data collection methods and flow of patients into study.

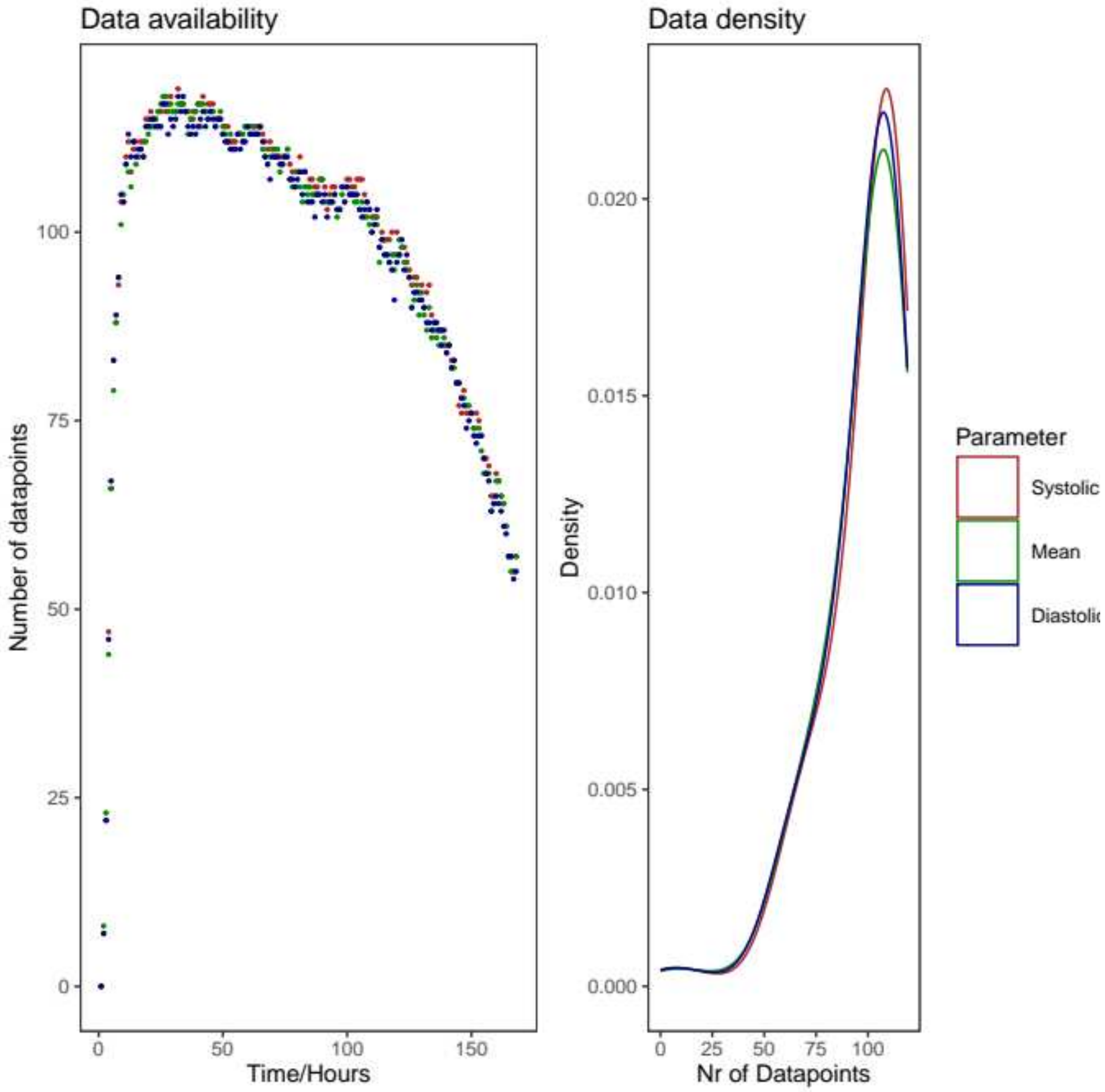

Supplementary Figure 2. Data availability per patient after outlier removal for the first 168 hours. Red indicates systolic data, green mean data, and blue diastolic data (left). Data density for the dataset,

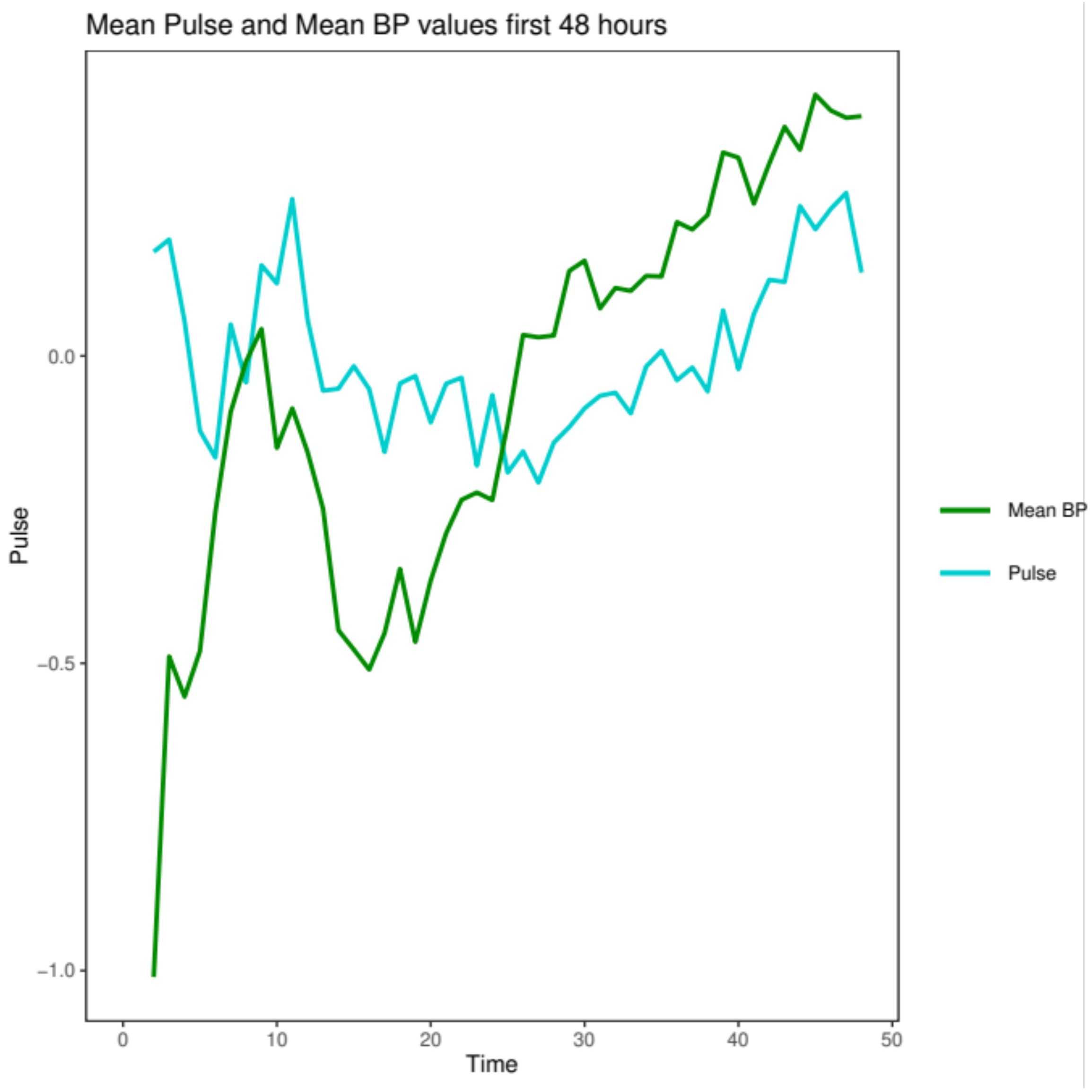

Supplementary Figure 3. Mean blood pressure and pulse rate for the first 48 hours, data has been scaled and centered to enable comparison of the respective dynamics.

Tables

Supplementary Table 1. Granger test statistics for comparison of various conditions. Significant test statistics (in both directions) in bold. Tests were performed with frequency=24 (i.e., one day) and lag=6.

Test	Parameter	F 1vs2	P-value 1vs2	F 2vs1	P-value 2vs1
Expired Vs Survived	S	0.7114	0.6411	1.7362	0.1181
	M	1.0648	0.3874	1.2985	0.2628
	D	1.0166	0.4177	2.7913	**0.01401 ***
IVH vs NoIVH	S	0.672	0.6725	1.3972	0.221
	M	1.8902	0.08768 .	0.7394	0.6189
	D	1.8553	0.09386 .	0.838	0.5429
NEC Vs NoNEC	*S*	1.0188	0.4163	2.2885	0.03959 *
	**M**	2.1973	**0.04762 ***	1.4941	0.1856
	D	2.2921	**0.0393 ***	4.1012	**0.0008705 *****
NEC And IVH	S	1.6417	0.1412	1.4911	0.1866
	M	1.1992	0.3112	1.1367	0.3451
	**D**	3.7469	**0.001853 ****	2.7138	**0.01647 ***
Week22 Vs Week 23	S	2.1184	0.05588 .	0.3234	0.9237
	M	3.7312	**0.001924 ****	1.7925	0.1061
	D	1.0904	0.372	2.2688	**0.04126 ***
Week22 Vs Week 24	S	1.598	0.1533	**1.4297**	0.2086
	M	0.6603	0.6818	1.1013	0.3656
	D	1.2781	0.2723	0.8565	0.5291
Week23Vs Week 24	S	6.5905	**4.651e-06 *****	**1.7821**	0.1081
	M	2.6125	**0.02034 ***	2.13	0.05452 .
	D	4.5404	**0.0003415 *****	2.0958	0.05839 .
Csec Vs Natural	S	3.9531	**0.001194 ****	2.2704	**0.04107 ***
	M	2.6132	**0.02031 ***	1.765	0.1117
	D	1.5071	0.1813	2.1612	0.05121 .
ChorioA Vs NoChorioA	S	3.2583	0.005236 **	1.0051	0.4252
	M	0.8812	0.5109	1.8826	0.08899 .
	D	2.337	0.03586 *	1.1103	0.3602

Supplementary Table 2. Output summary of generalized linear model for conditions and analyzed numerical variables. Model: IVH Grade ~ Week + Inotrope/vasoactive drug + NEC + >46mmHg at any time + %Time spent at>46mmHg + Nr.Fluctuations. Data were scaled and centered prior to analysis.

Condition	Estimate	Std. Error	z value	Pr(>|z|)	Sig Level
Week	-0.19562	0.09005	-2.172	0.029833	*
NECTRUE	-0.21959	0.28044	-0.783	0.433605	
GT46mmHgTRUE	0.59868	0.26861	2.229	0.025825	*
PercGT46mmHg	-0.18566	0.1585	-1.171	0.24146	
NrFluc168	-0.34582	0.09755	-3.545	0.000393	***

Supplementary Table 3. Outcome of Fisher's exact tests for patient outcome. Significant test statistics in bold, weakly significant test statistics in bold italics.

Test of proportions: Condition	Fisher's exact p-value	Odds Ratio	Sig. Diff.
Expired patients with IVH diagnosis Vs. No diagnosis	***0.0616***	***0.428422***	No
Expired patients with NEC diagnosis Vs. No diagnosis	**0.02968**	**0.3014454**	Yes
Expired patients with IVH&NEC diagnosis Vs. No diagnosis	**0.01159**	**6.036246**	Yes
IVH Diagnosis GA week 22 vs week 23	0.1812	0.2950553	No
IVH Diagnosis GA week 22 vs week 24	0.4857	0.4885168	No
IVH Diagnosis GA week 23 vs week 24	0.2462	1.656861	No
Patient Exceeded/Did not exceed 46mmHg and Expired	0.2567	1.735024	No
Patient Exceeded/Did not exceed 46mmHg and diagnosed with IVH	0.1698	1.833681	No
IVH Diagnosis Csec vs Norm	0.3632	0.6829341	No
IVH Diagnosis ChoriaA diagnosis vs No diagnosis	0.06121	0.4342773	No

Description of data extraction and statistical analyses

Data extraction, normalization, and removal of outliers

The output from the data monitoring systems in Microsoft Excel format were converted to TSV files and an in-house developed perl-script was used to average the values (recorded on a nominal one datapoint per minute but displaying gaps in the data) on a per-hour basis in order to compensate for gaps in the data. Furthermore, time elapsed since birth to beginning of monitoring was taken into account, resulting in normalized data comparable between patients.

Due to rapidly decreasing amount of data after the 168-hour mark (corresponding to approximately one week after birth), a cut-of at this hour was implemented and no later data-points were included in the analysis.

In order to account for spurious data, caused by measurement errors and other factors unlikely to have a biological/physiological basis, interquartile range criterion (IQR) was used to clean the data and remove outliers. This resulted in removal of 299, 602 and 535 datapoints for the systolic, mean and diastolic datasets, respectively.

Granger's causality tests for time-series data

Granger causality test is a statistical method used to determine whether one time series is a useful predictor of another time series. In other words, it can determine if a change in one variable can be used to predict the change in another variable. In the analysis presented here, it was used to determine if the BP curves were statistically similar enough that one curve could be used to predict another for a given set of conditions, i.e., if statistical similarity exist between BP development (e.g., inotrope/vasoactive drug having been given or not). If one curve is not able to predict the development of another, we can conclude that they are statistically significantly different, given that this is significant in both directions.

To account for the fact that the data was analysed in aggregate, i.e., the mean of all patients exhibiting a specific set of conditions, and that patients are likely to display variation in individual development, a frequency of 24 hours and a lag of 6 hours was used in the comparisons.

The “lmtest (version 0.9-40)” R package was utilized for all time-series analysis.

Effect of BP in specific “bands,” and identification of dangerous BP thresholds

The procedure described in Vesoulis *et al. w*as repeated for the time each patient spent in specific “bands,” corresponding to percent of total measurement time spent between specific defined thresholds of blood pressure.

However, no correlation between outcome (e.g., grade of IVH, patient expired) and percentage spent at specific BP values could be found in our data. However, a statistically significant difference was detected regarding if the patient at any time had exceeded the threshold identified by Vesoulis *et al.,* corresponding to 46mmHg for mean blood pressure.

Further, an attempt was made at identifying alternative thresholds with greater statistical effect than the threshold identified by Vesoulis *et al.* by receiver operating characteristic (ROC) analysis, but no threshold value with greater power could be detected.

Analysis of number of fluctuations in blood pressure, i.e., stableness

To analyse the number of fluctuations/how stable the blood pressure was for each patient, an analysis was performed where all patients with a maximum of half the time-points missing, and the missing time-points fixed by linear interpolation with the R na.interp function were included in the analysis. Subsequently, the number of times each patient blood pressure crossed its own 24-hour rolling mean was counted. This concept is illustrated for a singular patient in supplementary Figure 4, where the red line corresponds to the rolling mean and the black line corresponds to the actual BP values.

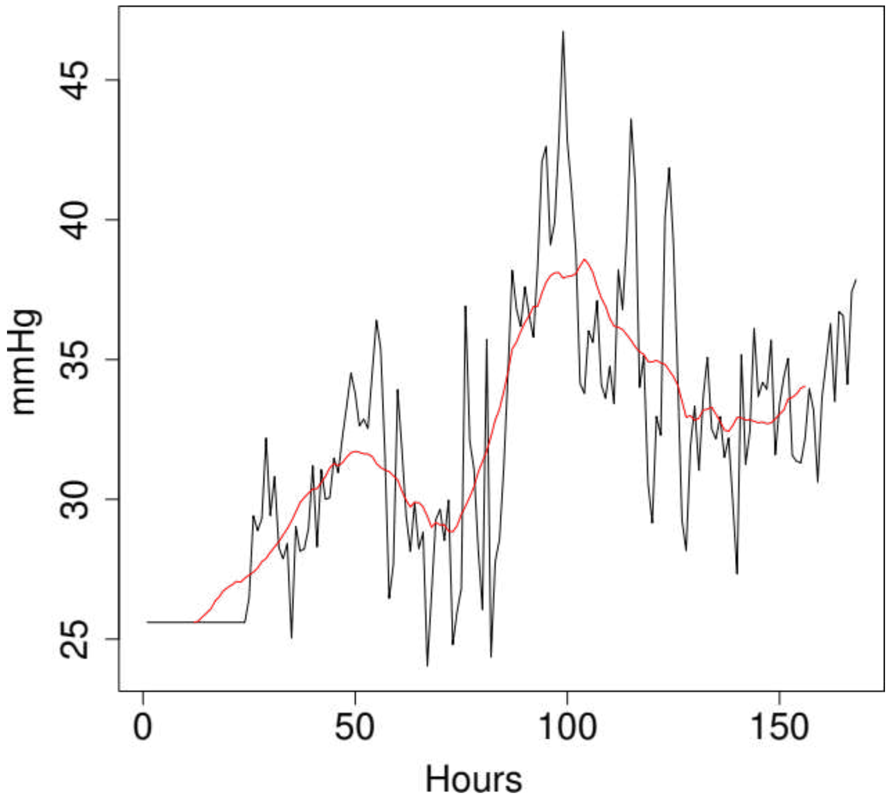

Supplementary Figure 4. Illustration of concept of analysis of number of fluctuations.

In other words, a numerical value was obtained for each patient measuring how many fluctuations took place around the rolling mean, giving a measurement indicating the how stable blood pressure is/slow response to divergent blood pressure.

Determination of nadir-point for individual patients

To detect the nadir point for individual patients, data-series with a maximum of 25% of the data-points missing were selected, and the R function cpt.mean from the "changepoint (version 2.2.4)" R package was used after linear interpolation with the R na.interp function to fix missing datapoints. In order to avoid spurious detections, the analysis was limited to the period between 8 and 36 hours. The parameters for cpt.mean were set as follows: penalty="MBIC",pen.value=0,method="AMOC",Q=5,test.stat="Normal",class=FALSE, param.estimates=TRUE,minseglen=1.

Evaluation of significant factors for inclusion in modelling

In order to evaluate potential factors to include in the general linearized model, a number of different models were evaluated, leading to the final model incorporating factors either previously described as being significant in the literature, or where a Fisher's exact test for our own data indicated that the outcome was numerically different, or where other analysis had suggested that explanatory power was present as in the case of number of fluctuations. A presentation of the factors evaluated can be found in Supplementary table 3. Akaike Information Criterion (AIC) was used to select the final model providing the best fit. The final model corresponded to: IVH Grade ~ Week + Inotrope/vasoactive drug + NEC + >46mmHg at any time + %Time spent at>46mmHg + Nr.Fluctuations.
