# Supplementary material for: Blood pressure trends following birth in infants born under 25 weeks’ gestational age: a retrospective cohort study
Source: BMJ Paediatr Open. 2024 Mar 25;8(1):e002438. doi: 10.1136/bmjpo-2023-002438 (PMC10966797; doi:10.1136/bmjpo-2023-002438)
Supplement: Author's manuscript [file bmjpo-2023-002438.draft_revisions.pdf]

BMJ Paediatrics Open

BMJ Paediatrics Open is committed to open peer review. As part of this commitment we make the peer review history of every article we publish publicly available.

When an article is published we post the peer reviewers' comments and the authors' responses online. We also post the versions of the paper that were used during peer review. These are the versions that the peer review comments apply to.

The versions of the paper that follow are the versions that were submitted during the peer review process. They are not the versions of record or the final published versions. They should not be cited or distributed as the published version of this manuscript.

BMJ Paediatrics Open is an open access journal and the full, final, typeset and author-corrected version of record of the manuscript is available on our site with no access controls, subscription charges or pay-per-view fees (http://bmjpaedsopen.bmj.com).

If you have any questions on BMJ Paediatrics Open's open peer review process please email info.bmjpo@bmj.com

BMJ Paediatrics Open

Blood pressure trends following birth in infants born under 25 weeks' gestational age: a retrospective cohort study

Journal:	*BMJ Paediatrics Open*
Manuscript ID	bmjpo-2023-002438
Article Type:	Original research
Date Submitted by the Author:	06-Dec-2023

Complete List of Authors:	Persad, Emma; Karolinska Institutet, Department of Women's and Children's Health; Karolinska University Hospital, Department of Neonatology<br>Brindefalk, Björn; Stockholm University, Department of Molecular Biosciences, The Wenner-Gren Institute<br>Rakow, Alexander; Karolinska Institutet, Department of Women's and Children's Health; Karolinska University Hospital, Department of Neonatology

Keywords:	Cardiology, Epidemiology, Neonatology

SCHOLARONE™
Manuscripts

I, the Submitting Author has the right to grant and does grant on behalf of all authors of the Work (as defined in the below author licence), an exclusive licence and/or a non-exclusive licence for contributions from authors who are: i) UK Crown employees; ii) where BMJ has agreed a CC-BY licence shall apply, and/or iii) in accordance with the terms applicable for US Federal Government officers or employees acting as part of their official duties; on a worldwide, perpetual, irrevocable, royalty-free basis to BMJ Publishing Group Ltd ("BMJ") its licensees and where the relevant Journal is co-owned by BMJ to the co-owners of the Journal, to publish the Work in this journal and any other BMJ products and to exploit all rights, as set out in our licence.

The Submitting Author accepts and understands that any supply made under these terms is made by BMJ to the Submitting Author unless you are acting as an employee on behalf of your employer or a postgraduate student of an affiliated institution which is paying any applicable article publishing charge ("APC") for Open Access articles. Where the Submitting Author wishes to make the Work available on an Open Access basis (and intends to pay the relevant APC), the terms of reuse of such Open Access shall be governed by a Creative Commons licence – details of these licences and which Creative Commons licence will apply to this Work are set out in our licence referred to above.

Other than as permitted in any relevant BMJ Author's Self Archiving Policies, I confirm this Work has not been accepted for publication elsewhere, is not being considered for publication elsewhere and does not duplicate material already published. I confirm all authors consent to publication of this Work and authorise the granting of this licence.

Confidential: For Review Only

Cover page

Full title

Blood pressure trends following birth in infants born under 25 weeks' gestational age: a retrospective cohort study

Confidential: For Review Only

Authors

Emma Persad[1,2]

Björn Brindefalk[3]

Alexander Rakow[1,2*]

Authors' institutional affiliations

[1]Department of Women's and Children's Health, Karolinska Institutet, Solna, Sweden

[2]Department of Neonatology, Karolinska University Hospital, Solna, Sweden

[3]Department of Molecular Biosciences, The Wenner-Gren Institute, Stockholm University, Stockholm, Sweden

Corresponding author

Alexander Rakow, Department of Women's and Children's Health, Karolinska Institutet, Tomtebodavägen 18A, 17177 Solna, Sweden, alexander.rakow@ki.se, +46725968444

Abstract

Objective

The aim of our study was to describe post-natal blood pressure (BP) trends and evaluate relevant dynamics and outcomes for a subgroup of extremely preterm (EPT) infants.

Design

Retrospective observational cohort study.

Setting

Patients admitted to Karolinska University Hospital Stockholm.

Patients

EPT infants born between 22+0 and 24+6 weeks' gestational age (GA) undergoing invasive, continuous BP monitoring through an umbilical arterial catheter.

Main outcome measures

Physiological BP trends, the influence of cardiovascular active interventions on BP, and relevant adverse outcomes were mapped over the first week of life.

Results

We included 125 infants between January 2009 and November 2021. Mean BP values were 31 mmHg, 32 mmHg, and 35 mmHg, at 3 hours, 24 hours, and 48 hours, respectively. A pronounced BP dip and nadir was observed around 15-20 hours. Thirty-six percent received cardiovascular active drugs, 48% developed intraventricular hemorrhage, 15% developed necrotizing enterocolitis, and 25% died.

Conclusions

The post-natal BP dip should be regarded as a physiological phenomenon and not automatic grounds for interventions which may momentarily stabilize BP but have no appreciable short- or long-term effects. Approximating clinically acceptable mean BP values using GA gives under-estimations in these infants. Further studies are warranted for improved understanding of clinically relevant trends and outcomes.

Confidential: For Review Only

What is already known on this topic

Low blood pressure (BP) affects 15-50% of extremely preterm (EPT) infants following birth and is associated with adverse outcomes, however, high-quality data outlining acceptable BP trends and accurate BP-estimation practices crucial for comprehensive patient monitoring are lacking in this cohort. Further, a post-natal BP dip has been observed in EPT infants although is not yet well-described or widely acknowledged, warranting clarification on the magnitude and timing of this dip and a high-quality descriptive analysis of BP to guide clinical care.

What this study adds

We provide valuable insight into BP trends observed in a typical group of 125 EPT infants born at the border of viability (22+0 to 24+6 weeks' gestational age [GA]), finding a significant drop in BP and a nadir reached at approximately 15-20 hours after birth, regardless of cardiovascular active drug administration. Additionally, we show that methods for predicting BP based on GA significantly underestimate the actual BP levels in this cohort.

How this study might affect research, practice or policy

Clinical guidelines and practitioners should recognize the post-natal BP dip as a physiological occurrence, avoid GA-based BP estimation practices, acknowledge daily increments of BP, and reconsider the routine use of cardiovascular active drugs during the post-natal period to stabilize assumed hypotensive episodes, instead utilizing additional indicators of perfusion to access hemodynamic stability.

Confidential: For Review Only

INTRODUCTION

Despite increasing ability to care for extremely preterm infants (EPT), defined as those born under 28 weeks' gestational age (GA), considerable uncertainty surrounding age- and condition-appropriate blood pressure (BP) remains.(1-6) Furthermore, data on the efficacy of anti-hypotensive treatment and relevant thresholds to initiate treatment are lacking, despite episodes of assumed hypotension often routinely being targeted with cardiovascular active drugs or volume boluses potentially associated with increased morbidity.(7, 8) Comparatively, not intervening during hypotensive episodes can be equally harmful, particularly for neurodevelopment.(9-11) Clinical management of these infants is further complicated by their rapidly-evolving physiology and numerous medical interventions, which may influence or mask natural BP trends.(12)

Neonatal intensive care units (NICUs) care for an increasing number of EPT infants prone to severe outcomes and complications, such as intraventricular hemorrhage (IVH), necrotizing enterocolitis (NEC), and early neonatal death.(4, 5) There are limited high-quality BP data on infants under 25 weeks' GA, particularly as very few centers admit infants this young or have access to comprehensive databases and continuous monitoring data. Consequently, clinical treatment is informed by outdated and error-prone extrapolated data from older infants.(2)

A post-natal BP dip and immaturity-related BP fluctuations have been observed in EPT infants, although a comprehensive exploration of these phenomena and their long-term implications are not well-described.(3, 13) Despite these BP variations being reported in the literature, clinicians commonly follow the consensus-based practice of estimating acceptable mean BP using the infants' GA in weeks.(14, 15) Hypotension in EPT infants has also been defined as mean arterial pressure <30 mmHg or less than the 10th percentile of gestational and postnatal age from published norms, which are often based on non-invasive measurements prone to over-estimations and

Confidential: For Review Only

artifacts.(2, 3, 16) As providing evidence-based, high-quality care is crucial for these vulnerable infants and assumed hypotensive episodes often prompt invasive interventions to stabilize BP, clarification on age-appropriate BP and the magnitude and timing of the post-natal dip is warranted for improved clinical management. This study aims to describe BP in EPT infants born before 25 weeks' GA in our single-center cohort, providing insight into the physiological development and outcomes of a typical group of vulnerable, underdeveloped patients subject to multiple supportive interventions.

METHODS

This was a retrospective observational cohort study in EPT infants admitted to Karolinska University Hospital Stockholm between January 2009 and November 2021. We included all infants born between 22+0 and 24+6 weeks' GA with an umbilical arterial catheter enabling continuous invasive BP monitoring recorded at one-minute intervals. Infants over 25+0 weeks' GA, without invasive monitoring, and with chromosomal abnormalities were excluded (Supplementary Figure 1).

Patient Monitoring Solutions (Clinisoft GE®) was used to identify eligible patients using both GA and Diagnosis Related Group codes and retrieve monitoring data for all patients to limit selection bias. Patient documentation systems (TakeCare® and Obstetrix®) were used to extract demographic information and outcomes. All non-anonymized data were stored on Region Stockholm servers and not shared externally.

Data were extracted for BP values (systolic, mean, diastolic), time between birth and monitoring, incidence and time of IVH, NEC, and death, and demographic factors, including GA (weeks + days), time of birth, weight (grams), vasopressor or inotropic drugs (dopamine and dobutamine in

Confidential: For Review Only

this cohort), respiratory support, mode of delivery, maternal steroids, and maternal chorioamnionitis.

We normalized the time elapsed from birth until start of invasive measurement, aggregating data on a per-hour basis to account for un-even sampling (in-house Perl script available on request). We focused our investigation between hours 1 and 168 of life, corresponding to a maximum of 76 million per-minute measurements, resulting in 16555, 16390, and 16354 data points following interquartile range-based outlier removal for systolic, mean, and diastolic BP values, respectively (Supplementary Figure 2).

All statistical testing and data-handling apart from initial extraction was done with R Statistical Software (version 4.0.3).(17) We evaluated statistically significant differences in time-series using Granger's causality tests with bidirectional significance indicating similarity between curves. Modelling of significant factors for IVH severity was done with a generalized linear model (GLM) where numerical variables were centered and re-scaled and other variables treated as factors (Model: IVH Grade ~ Week + Inotrope/vasoactive drug + NEC + >46mmHg at any time + %Time spent at>46mmHg + Nr.Fluctuations). For further analysis details, see supplementary material. We did not involve patients or the public in the design, conduct, reporting, or dissemination plans.

RESULTS

Demographics

This study included 125 infants born below 25 weeks' GA at Karolinska University Hospital Stockholm between 2009 and 2021. Our cohort comprised 11, 60, and 54 infants born at week 22, 23, and 24, respectively. Table 1 outlines baseline demographic data.

Table 1.

Blood pressure trends during the first 48 hours

Confidential: For Review Only

Increasing BP trends were observed over time. Our cohort had a mean BP of 31 mmHg at baseline (3 hours after birth), 32 mmHg at 24 hours (1 mmHg increase from baseline), and 35 mmHg at 48 hours (4 mmHg increase from baseline). Average systolic, mean, and diastolic BP values and incremental changes from baseline are presented in Table 2.

Table 2.

Figure 1a illustrates mean BP trends for all infants with the 25th and 75th quartiles, showing an initial increase and subsequent decrease during the first 24 hours. Figure 1b presents simplified findings suitable for clinical use.

Figure 1.

Figure 2 illustrates BP trends for all infants during the first 48 hours with a pronounced dip and nadir observed 15-20 hours following birth.

Figure 2.

Differences in blood pressure trends for various conditions

Granger's continuity tests showed significantly different BP trends when considering aggregate data for most conditions (Supplementary Table 1). A refined representation of BP trends for infants in the three GA cohorts and plots comparing infants that died vs. survived, developed IVH or NEC vs. did not, and received drugs vs. did not during the first 168 hours of life are shown in Figure 3 (Supplementary Table 3).

Figure 3.

Model analysis and correlation of various factors to IVH

Using a GLM, we tested time spent in BP-bands >46 mmHg or <23 mmHg to evaluate potential correlations with IVH grade as reported by Vessoulis *et al*, however found no such correlations (Supplementary Table 1, Supplementary Figure 3).(13) Interestingly, we found a significant

Confidential: For Review Only

correlation between infants exceeding 46 mmHg at least once and increased severity of IVH ($p=0.02$). To evaluate BP fluctuations, we calculated the number of the times BP crossed its own 24-hour moving average for a numerical measurement of BP stability, finding that a slow response to divergent BP was strongly correlated to increased IVH severity ($p\leq0.001$). We also observed a statistically significant correlation between severity of IVH grade and lower GA ($p=0.03$).

DISCUSSION

To our knowledge, this is the first description of continuous invasively measured BP data in infants born under 25 weeks' GA, providing insight into an extremely vulnerable population for which data is often error-prone and extrapolated. Between 2009 and 2021, the clinical management of EPT infants at our center, comprising ventilation strategies, fluid and bolus management, inotropic use, and tolerance of hypercapnia, saw minimal changes. This stability can be attributed to our center's conservative approach, emphasizing the use of established and reliable methodologies. Notably, our ventilation strategy has remained unchanged since 2003, and the array of cardiovascular active drugs administered has not altered significantly. The only noteworthy modifications include the admission of infants born at 22 weeks' GA from 2016 and an increased emphasis on delayed cord clamping from 2018, meaning the overall management framework has adhered to a rigid, time-tested paradigm. As interventions to address suspected hypotensive BP can vary across countries and medical centers, our single-center perspective provides a clear and consistent snapshot of our institution's practices and enhances the robustness of our findings.

Our cohort illustrated non-linear BP dynamics, finding a decrease in BP following birth with a nadir at approximately 15-20 hours (Figure 2; Supplementary Figure 4). A markedly later nadir was also observed for the 22 weeks' GA cohort, although this was not significant and was more pronounced for diastolic BP (Supplementary Figure 4). We also observed significant differences

Confidential: For Review Only

in BP trends when comparing infants born at 22, 23, or 24 weeks' GA, highlighting the potential effect of immaturity on physiology, even for only a 1-2 week difference. Although there were far fewer week 22 infants overall, the differences in BP trends between GA groups further supports the later nadir exhibited in these infants (Supplementary Table 2). Comparatively, a nadir at approximately 4-5 hours following birth has been reported in infants aged 23-25 weeks' GA, although BP was recorded non-invasively, leading to potential inaccuracies.(3) As our cohort exclusively received invasive BP measurements, the timing and dip in BP is seemingly physiological and not attributable to measurement error, which is of immense clinical relevance as it often coincides with the initiation of anti-hypotensive treatment. Although the physiology behind this dip remains unclear, it is proposed to be linked to cardiac output, which has exhibited a similar dip 6-12 hours following birth in older preterm infants, and neuroendocrine factors, such as catecholamines and hormones.(18-21) Identifying the mechanisms behind this physiological change and influence of GA on overall trends is crucial for a better understanding of EPT infants' natural post-natal physiology and development.

Our findings add to the literature challenging the practice of GA-based estimation of post-natal BP in EPT infants.(13) In our cohort, infants born between 22+0 and 24+6 weeks' GA had a mean BP of 31 mmHg at 3 hours, 32 mmHg at 24 hours, and 35 mmHg at 48 hours, considerably higher than estimated using GA-based practices. Our findings were similar to Vesoulis *et al.*, who estimated mean BP in infants <28 weeks' GA as 33 mmHg following birth.(13) Similarly, Kent *et al.* reported a mean BP range of 31-43 mmHg at 24 hours in infants aged 28-29 weeks' GA.(16) This encompasses our findings of 32 mmHg, although their measurements were recorded non-invasively, potentially explaining their high upper range of 43 mmHg. Similarly, Batton *et al.* reported a BP increase of 0.2 mmHg per hour, an increase which our cohort did not exhibit;

Confidential: For Review Only

however, they did not strictly use invasive measurement techniques, which remain the most reliable means of BP monitoring.(3)

Although mean BP trends were different in infants that received cardiovascular active drugs vs. those who did not, our tests did not show that BP was lower or higher in either group overall. We complemented this with t-tests to compare time-specific BP levels, finding that average mean BP was significantly different between the two groups at 36 hours but not at 150 hours (Supplementary Table 4). Further, of the infants developing IVH, 38% had received cardiovascular active drugs, indicating that drugs may momentarily decrease BP fluctuations, however, have limited effect on the physiological nadir following birth, eventual BP level, and overall outcomes. Further research evaluating their effect on BP fluctuations, appropriate times for administration, and relevant outcomes in a larger cohort is warranted.

Different mean BP trends were found between major comparisons, indicating that eventual death, IVH diagnosis alone, IVH and NEC diagnosis combined, GA, cardiovascular active drug administration, maternal factors, and delivery method may be correlated to mean BP trends or influence mean BP enough to alter trends when compared to infants in the respective comparison groups (Supplementary Table 1). However, these tests only revealed differences in trends and not that one trend could be connected to specific outcomes, and the findings were only evident when considering aggregate data. Further exploration is required to draw more in-depth conclusions.

We found no correlation between time spent in specific BP-bands suggested as thresholds (>46 mmHg and <23 mmHg) and eventual IVH diagnosis.(13) However, infants who had crossed 46 mmHg at any time were significantly correlated to more severe IVH grade. Further, our data did not show the same correlation between increased instability and increased severity of IVH

Confidential: For Review Only

observed by Vesoulis *et al*., but instead found that a slow response to divergent BP was strongly correlated to increased IVH severity. However, cohort differences and Vesoulis *et al*. not detailing their statistical analysis rendered a direct comparison difficult.

While guidelines outlining thresholds for intervention would be clinically useful, our analyses highlight that the complexity of BP trends renders numerical values difficult to generalize and utilize. Retrospectively analyzing BP trends over time may display patterns indicating a risk for adverse outcomes, however, apart from having crossed 46 mmHg, we could not identify stringent numerical limits to define clinically safe BP thresholds. Although clinicians are particularly inclined to intervene to stabilize BP in EPT infants, our data showed a seemingly natural dip following birth and no correlation between low BP and adverse outcomes.(13, 16) If regarded as a natural physiological reaction, this dip should not be automatic grounds for systemic intervention, which may even be counter-productive considering the potential risks. Thus, it is inadequate to rely solely on BP to assess hemodynamic stability in clinical practice and imperative to utilize additional indicators of perfusion before commencing interventions.

This study provides quantitative and qualitative improvements to the literature, namely the high-quality continuous data in a comparatively large infant cohort for this GA group. Our cohort also had a narrow GA range, allowing us to infer trends representative of this understudied population. However, our results were limited by only evaluating 11 infants born in week 22. Although this is novel for the literature, the findings for this subset should be viewed with caution. It is unclear whether the lesser fluctuations observed in this group are due to immaturity-related physiological factors or low patient numbers. Further, 93% of infants were on HFOV, as this our first line ventilation strategy for EPT infants, which has the potential to affect preload and afterload and

Confidential: For Review

thereby cardiac output, subsequently decreasing BP when high mean airway pressures are used. We also did not correlate data on cord clamping procedures or administration of fluid boluses and blood transfusions, however, it can be assumed that most infants in this age cohort receive these interventions routinely and thus, these results are still indicative of this population. Nevertheless, these values should be regarded as approximate guidelines when treating infants in this age range.

CONCLUSION

This study presents highly accurate, invasive BP data on a typical cohort of unstable EPT infants born under 25 weeks' GA. Our findings highlight the post-natal BP dip as a physiological phenomenon and not immediate grounds for medical intervention provided other indicators of perfusion are promising, as cardiovascular active drugs may stabilize BP momentarily but have no appreciable short- or long-term effects. Our results also challenge the GA-based practice of estimating mean BP, encouraging clinicians to reconsider this rule and acknowledge its vast underestimation in these infants. Overall, our data did not reveal specific BP trends to be correlated with poor outcomes, challenging the use of BP as a sole indicator of hemodynamic stability and warranting future investigations. Further research using invasive data on larger cohorts of EPT infants is necessary to clarify acceptable physiological BP trends to optimize clinical care.

Confidential: For Review Only

Acknowledgements

We would like to thank Raman Abram for his technical support and guidance with the patient databases.

Competing interests

The authors have no potential conflicts of interest to report.

Funding

No funding was secured for this study.

Contributors

EP made substantial contributions to the design and acquisition of data, drafted the article and revised it critically for important intellectual content, and approved the final version to be published.

BB made substantial contributions to the analysis and interpretation of data, revised the article critically for important intellectual content, and approved the final version to be published.

AR made substantial contributions to the conception and design of the study, interpreted the data, drafted the article and revised it critically for important intellectual content, and approved the final version to be published.

Research Ethics Approval

Ethical approval was obtained from the Swedish Ethical Review Authority (Etikprövningsmyndigheten, approval number: 2022-01588-01-262498).

Patient consent for publication

Not applicable

Data availability statement

Confidential: For Review Only

The data supporting the findings of this study (deidentified participant data) are available upon reasonable request to the corresponding author.

References

1. Kent AL, Meskell S, Falk MC, Shadbolt B. Normative blood pressure data in non-ventilated premature neonates from 28-36 weeks gestation. Pediatr Nephrol. 2009;24(1):141-6.
2. Miall-Allen VM, de Vries LS, Whitelaw AG. Mean arterial blood pressure and neonatal cerebral lesions. Arch Dis Child. 1987;62(10):1068-9.
3. Batton B, Li L, Newman NS, Das A, Watterberg KL, Yoder BA, et al. Evolving blood pressure dynamics for extremely preterm infants. J Perinatol. 2014;34(4):301-5.
4. Development of audit measures and guidelines for good practice in the management of neonatal respiratory distress syndrome. Report of a Joint Working Group of the British Association of Perinatal Medicine and the Research Unit of the Royal College of Physicians. Arch Dis Child. 1992;67(10 Spec No):1221-7.
5. Pejovic B, Peco-Antic A, Marinkovic-Eric J. Blood pressure in non-critically ill preterm and full-term neonates. Pediatr Nephrol. 2007;22(2):249-57.
6. Batton B, Batton D, Riggs T. Blood pressure during the first 7 days in premature infants born at postmenstrual age 23 to 25 weeks. Am J Perinatol. 2007;24(2):107-15.
7. Bakshi S, Koerner T, Knee A, Singh R, Vaidya R. Effect of Fluid Bolus on Clinical Outcomes in Very Low Birth Weight Infants. J Pediatr Pharmacol Ther. 2020;25(5):437-44.
8. Joynt C, Cheung PY. Treating Hypotension in Preterm Neonates With Vasoactive Medications. Front Pediatr. 2018;6:86.
9. Fanaroff AA, Fanaroff JM. Short- and long-term consequences of hypotension in ELBW infants. Semin Perinatol. 2006;30(3):151-5.
10. Verma RP, Dasnadi S, Zhao Y, Chen HH. Complications associated with the current sequential pharmacological management of early postnatal hypotension in extremely premature infants. Proc (Bayl Univ Med Cent). 2019;32(3):355-60.
11. Schwepcke A, Weber FD, Mormanova Z, Cepissak B, Genzel-Boroviczeny O. Microcirculatory mechanisms in postnatal hypotension affecting premature infants. Pediatr Res. 2013;74(2):186-90.
12. Zubrow AB, Hulman S, Kushner H, Falkner B. Determinants of blood pressure in infants admitted to neonatal intensive care units: a prospective multicenter study. Philadelphia Neonatal Blood Pressure Study Group. J Perinatol. 1995;15(6):470-9.
13. Vesoulis ZA, El Ters NM, Wallendorf M, Mathur AM. Empirical estimation of the normative blood pressure in infants <28 weeks gestation using a massive data approach. J Perinatol. 2016;36(4):291-5.
14. van Zadelhoff AC, Poppe JA, Willemsen S, Mauff K, van Weteringen W, Goos TG, et al. Age-dependent changes in arterial blood pressure in neonates during the first week of life: reference values and development of a model. Br J Anaesth. 2023;130(5):585-94.

Confidential: For Review Only

15. Stranak Z, Semberova J, Barrington K, O'Donnell C, Marlow N, Naulaers G, et al. International survey on diagnosis and management of hypotension in extremely preterm babies. Eur J Pediatr. 2014;173(6):793-8.
16. Kent AL, Kecskes Z, Shadbolt B, Falk MC. Normative blood pressure data in the early neonatal period. Pediatr Nephrol. 2007;22(9):1335-41.
17. R. R; A language and environment for statistical computing: R Foundation for Statistical Computing; 2020 [Available from: https://www.R-project.org/.
18. Banerjee. Non-invasive continuous measurement of cardiac output in neonatal intensive care using regional impedence cardiography. Royal Society of Medicine2022. p. 11.
19. Ezaki S, Suzuki K, Kurishima C, Miura M, Moriwaki K, Arakawa H, et al. Levels of catecholamines, arginine vasopressin and atrial natriuretic peptide in hypotensive extremely low birth weight infants in the first 24 hours after birth. Neonatology. 2009;95(3):248-55.
20. Johansson S, Norman M, Legnevall L, Dalmaz Y, Lagercrantz H, Vanpee M. Increased catecholamines and heart rate in children with low birth weight: perinatal contributions to sympathoadrenal overactivity. J Intern Med. 2007;261(5):480-7.
21. Murase M, Ishida A, Momota T. Serial pulsed Doppler assessment of early left ventricular output in critically ill very low-birth-weight infants. Pediatr Cardiol. 2002;23(4):442-8.

Tables

Table 1. Patient demographics, parameters, and outcomes.

Demographics (125 infants)	n /data (%)
22 weeks' GA	11 (9%)
23 weeks' GA	60 (48%)
24 weeks' GA	54 (43%)
Average weight	627 g
Average time to invasive BP monitoring	222 mins – 3 hrs 42 mins
HFOV received	116 (93%)
Cardiovascular active drugs received	45 (36%)
IVH any	60 (48%)
IVH Grade I+II / III+IV	45 (75%) / 15 (25%)
Cardiovascular active drugs received + developed IVH	23/60 (38%)
NEC	19 (15%)
NEC operated	18/19 (95%)
Death overall	31 (25%)
Death within 2 weeks	13 (10%)
Average age at death overall	35 days
Average age at death if died within 2 weeks	5 days
Vaginal delivery	70 (56%)

Confidential: For Review Only

Mother given antenatal steroids	108 (86%)
Chorioamnionitis present	31 (25%)

Table 2. Average systolic (S), mean (M), and diastolic (D) blood pressure (mmHg) and incremental change data for all 125 infants over time.

	Blood pressure (mmHg)		
**Time**	**S**	**M**	**D**
3 hours	39	31	24
12 hours	40	33	26
24 hours	42	32*	25*
36 hours	44	34	26
48 hours	46	35	27
Increase from 3 hours to 12 hours	1	1	2
Increase from 3 hours to 24 hours	3	1	1*
Increase from 3 hours to 36 hours	6	3	2
Increase from 3 hours to 48 hours	7	4	2
Change between 3 hours and 24 hours	3	1	1
Change between 24 hours and 48 hours	4	3	1

* Indicates a decrease in mmHg between 12 hours and 24 hours

Confidential: For Review Only

Figures

Confidential: For Review Only

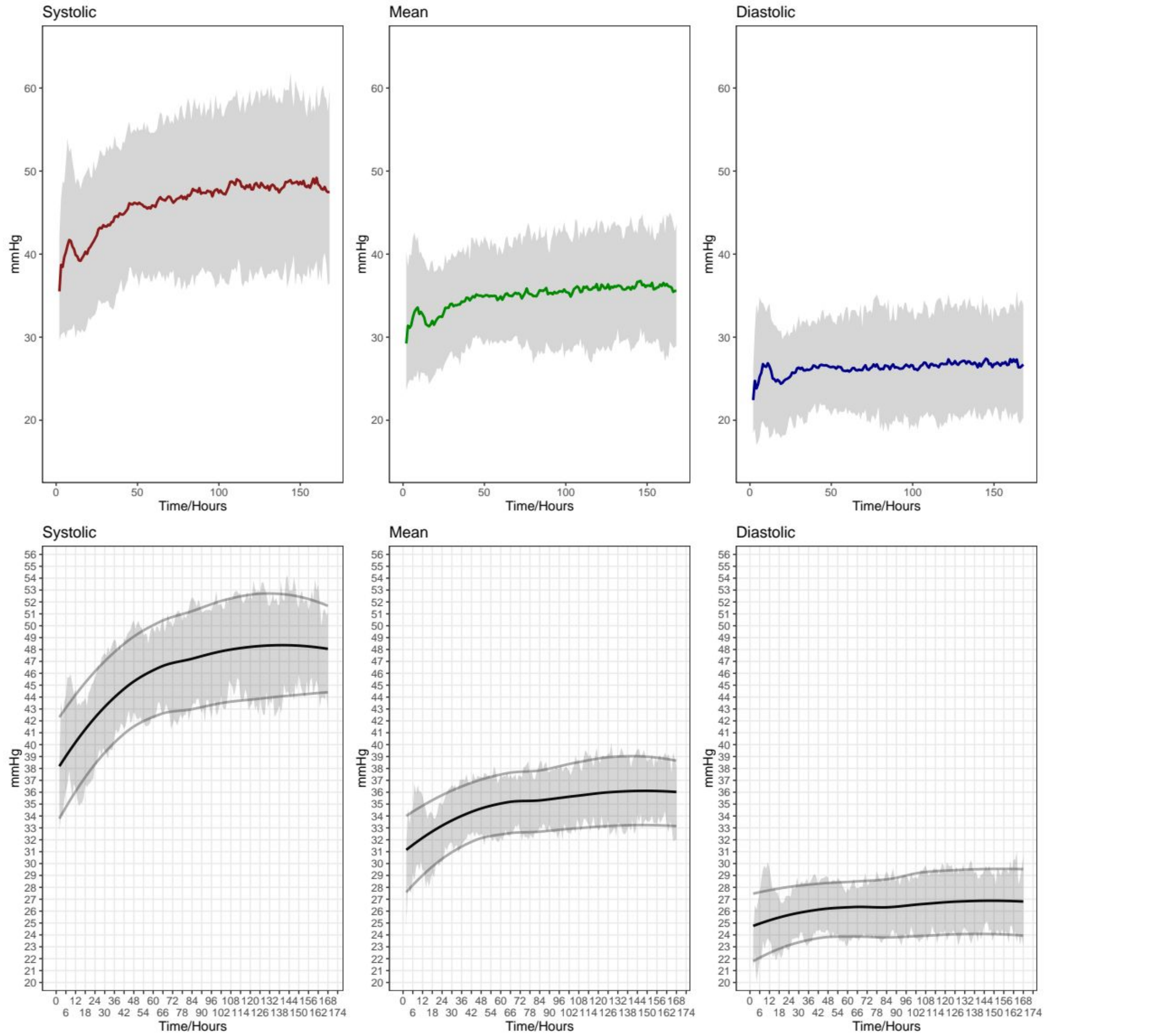

Figure 1a. Presentation of total data for 125 infants; mean value and quartiles (shaded area) for systolic (red), mean (green), and diastolic (blue) values for hours 1-168. Shading corresponds to first and third quartile for each dataset. **1b.** Plots showing the smoothed quintiles and mean value for systolic, mean, and diastolic data for the first 168 hours, giving a simplified summary of expected BP trends for extremely premature babies. Curves were smoothed with the Loess function and span=0.7 for easier interpretation.

Only

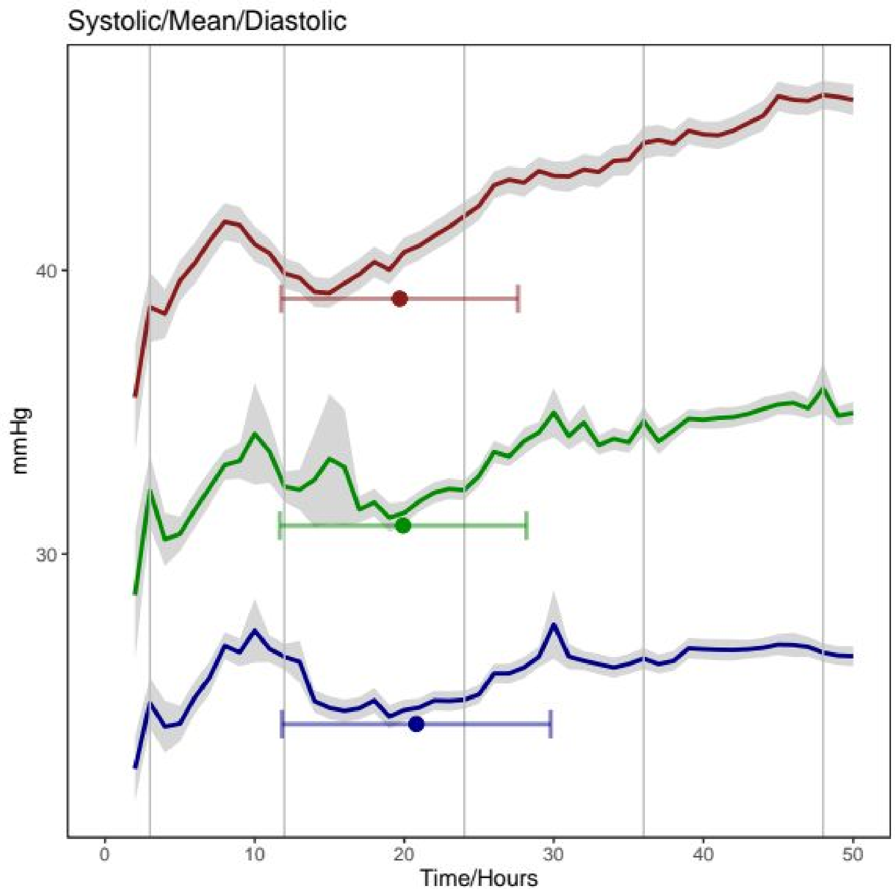

Figure 2. Detailed plot showing BP trends for all infants during the first 48 hours after birth with vertical lines corresponding to the time frames 3-12h, 12-24h, 24-36h and 36-48h). Shading corresponds to first and third quartile for each dataset. The error-bars indicate the mean nadir point and standard deviation for the nadir point for each dataset.

For Peer Review Only

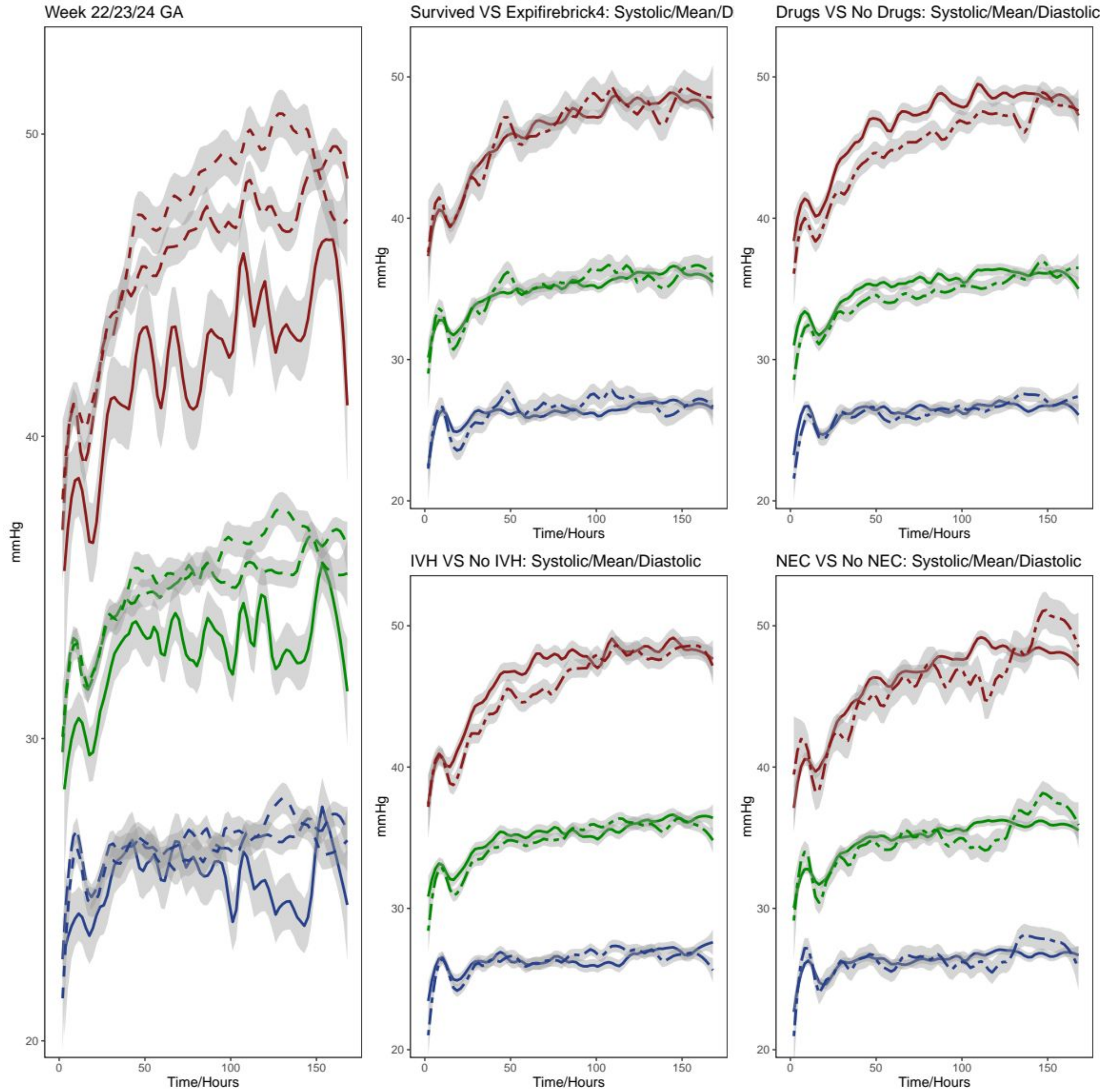

Figure 3. Comparison of systolic (red), mean (green), and diastolic (blue) BP trends over time for different week cohorts. Solid lines correspond to week 22, long dashes week 23, and short dashes week 24 GA (left). Solid line indicates base condition (survived, no drugs, no diagnosis) while dashed line indicates the alternative (right). Curves were smoothed with the Loess function and span=0.1.

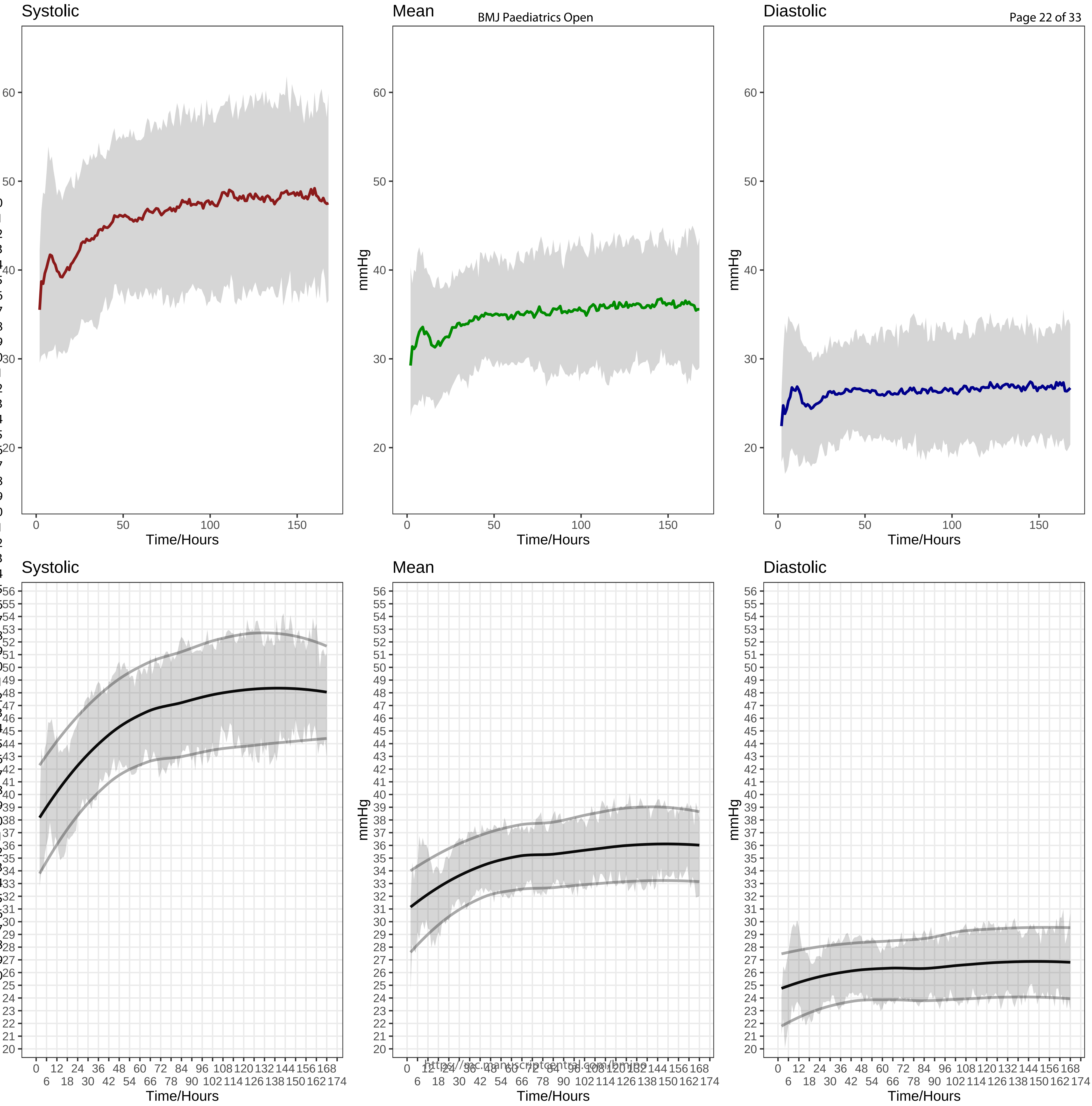
Systolic
Mean
Diastolic
mmHg
Time/Hours
Systolic
Mean
Diastolic
mmHg
Time/Hours

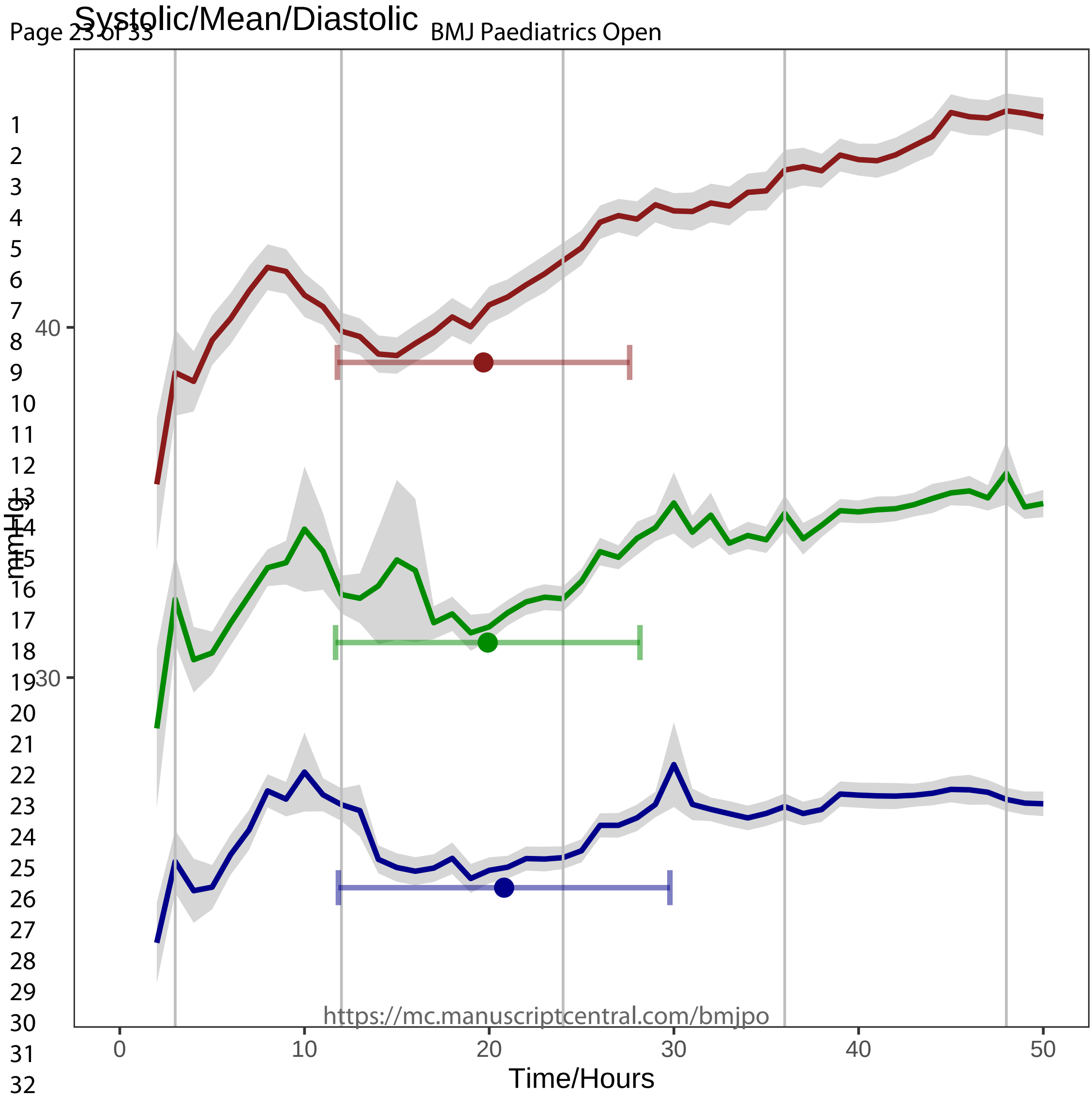
Systolic/Mean/Diastolic
mmHg
40
30
0
10
20
30
40
50
Time/Hours

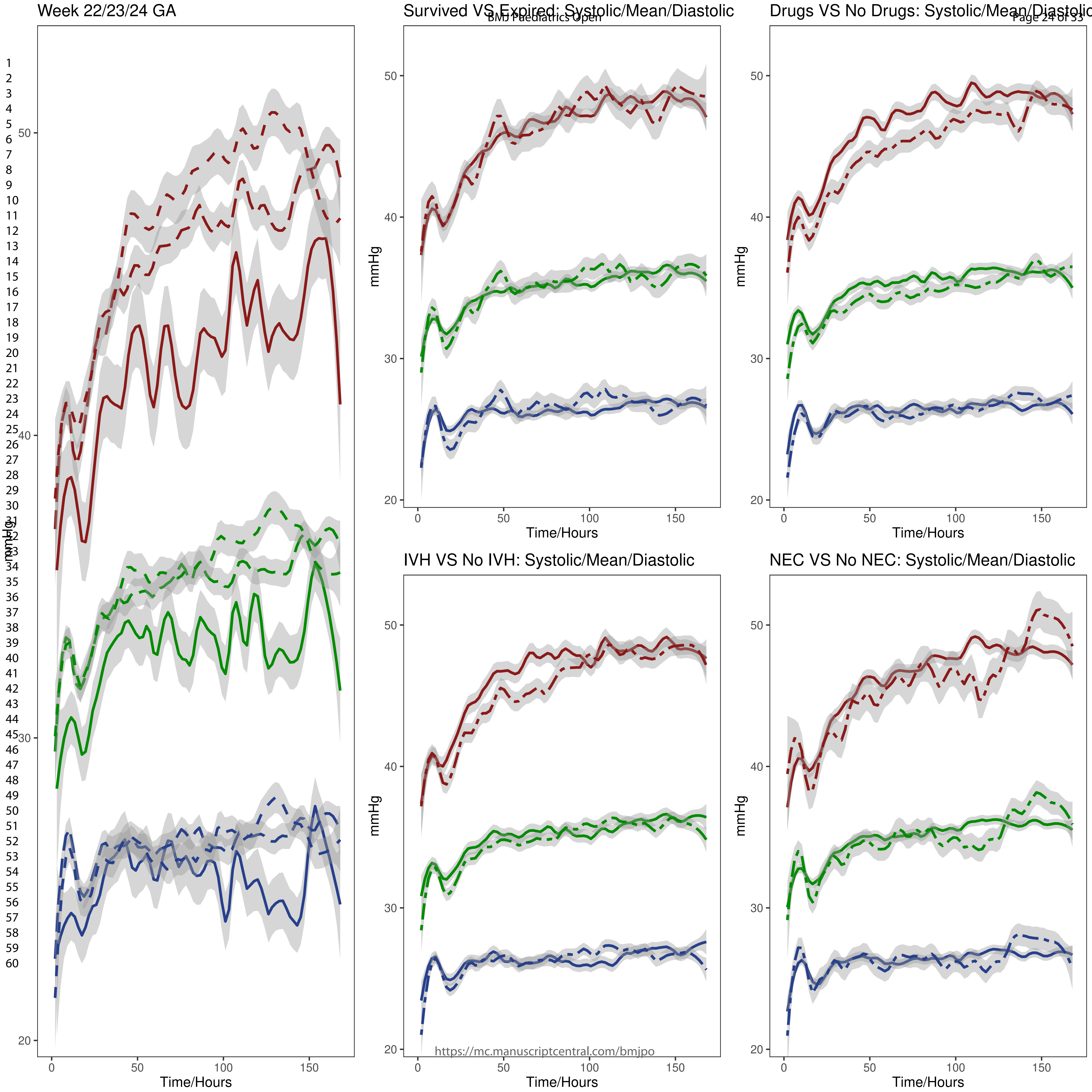
Week 22/23/24 GA
Survived VS Expired: Systolic/Mean/Diastolic
Drugs VS No Drugs: Systolic/Mean/Diastolic
IVH VS No IVH: Systolic/Mean/Diastolic
NEC VS No NEC: Systolic/Mean/Diastolic
mmHg
Time/Hours
20
30
40
50
0
50
100
150

Blood pressure trends following birth in infants born under 25 weeks' gestational age - Supplementary Material

Emma Persad, MD, Björn Brindefalk, PhD, Alexander Rakow, MD, PhD

Figures

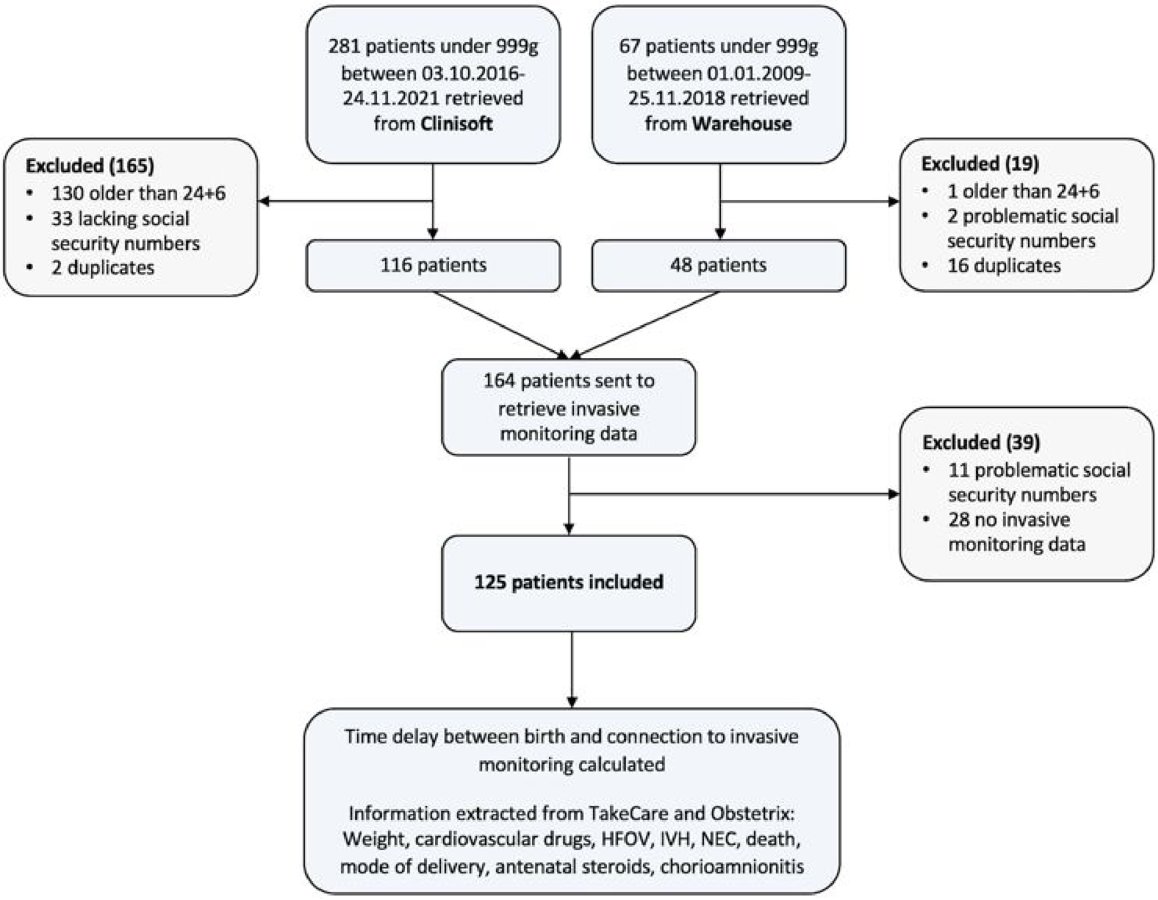

Supplementary Figure 1. Data collection methods and flow of patients into study.

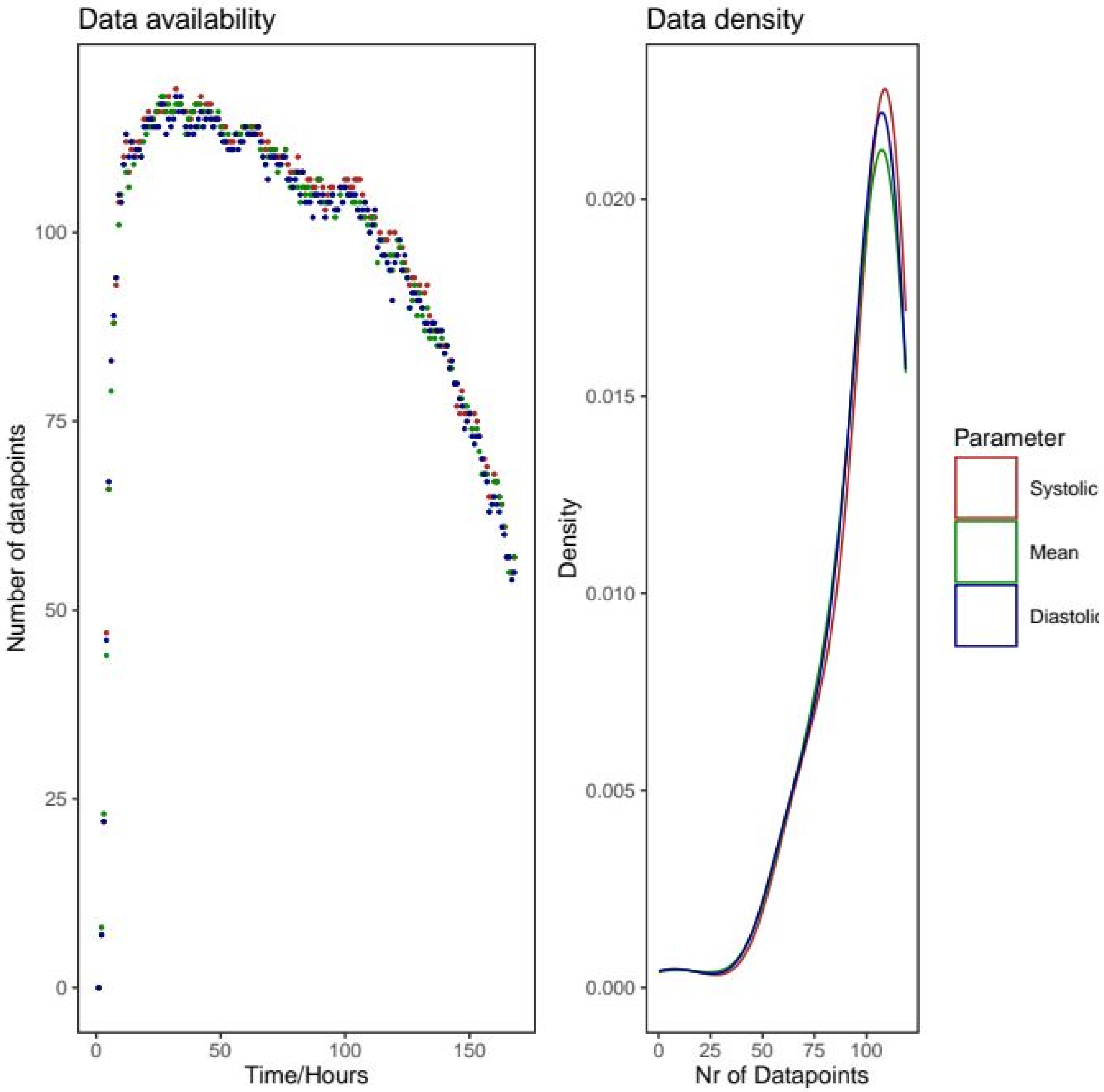

Supplementary Figure 2. Data availability per patient after outlier removal for the first 168 hours. Red indicates systolic data, green mean data, and blue diastolic data (left). Data density for the dataset, color coding as per panel (right).

Only

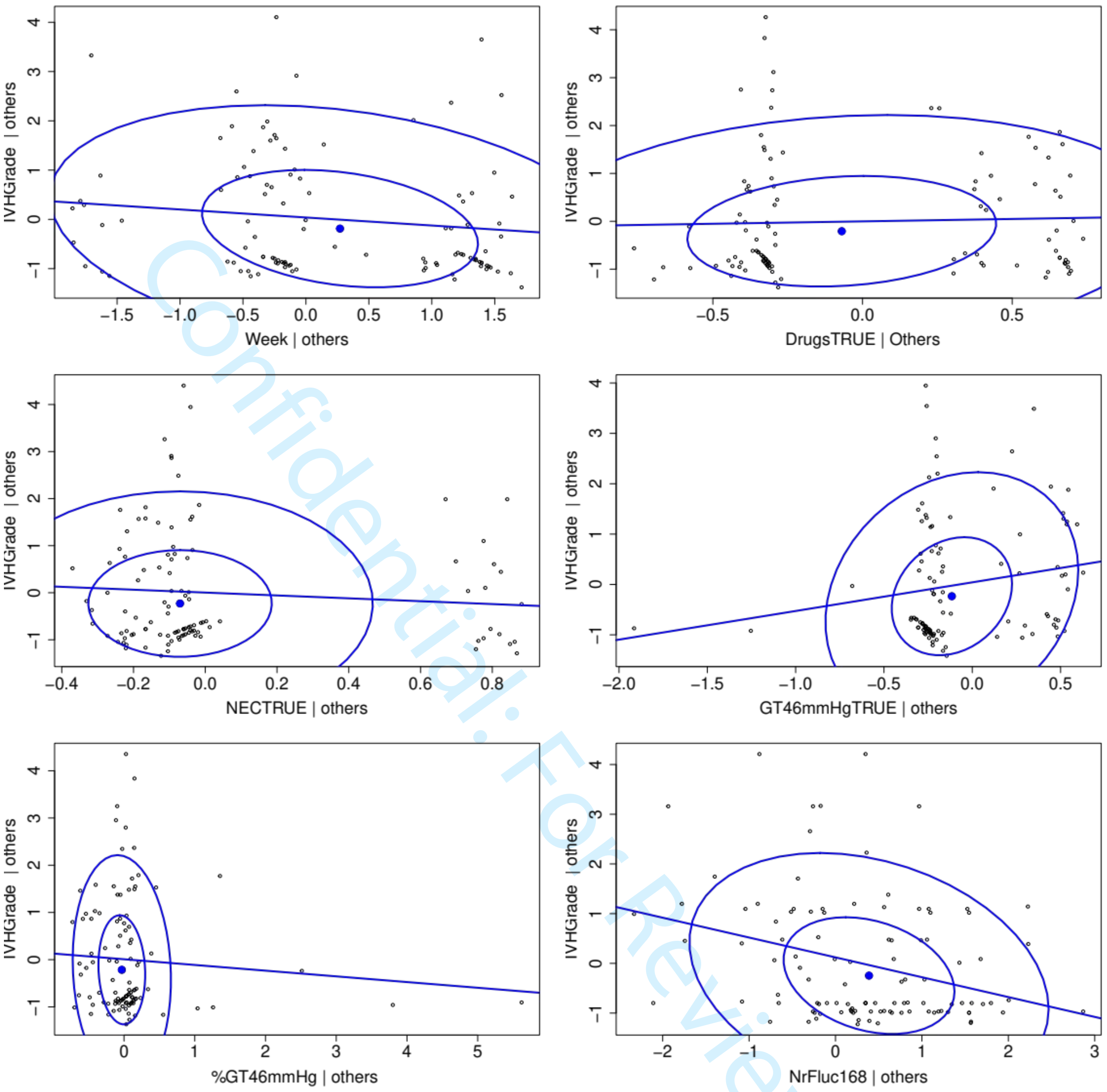

Supplementary Figure 3. Added-variable plot for conditions included in the generalized linear model: IVH Grade ~ Week + Inotrope/vasoactive drug + NEC + >46mmHg at any time + %Time spent at>46mmHg + Nr.Fluctuations. Data were scaled and centered prior to analysis.

Confidential: For Review Only

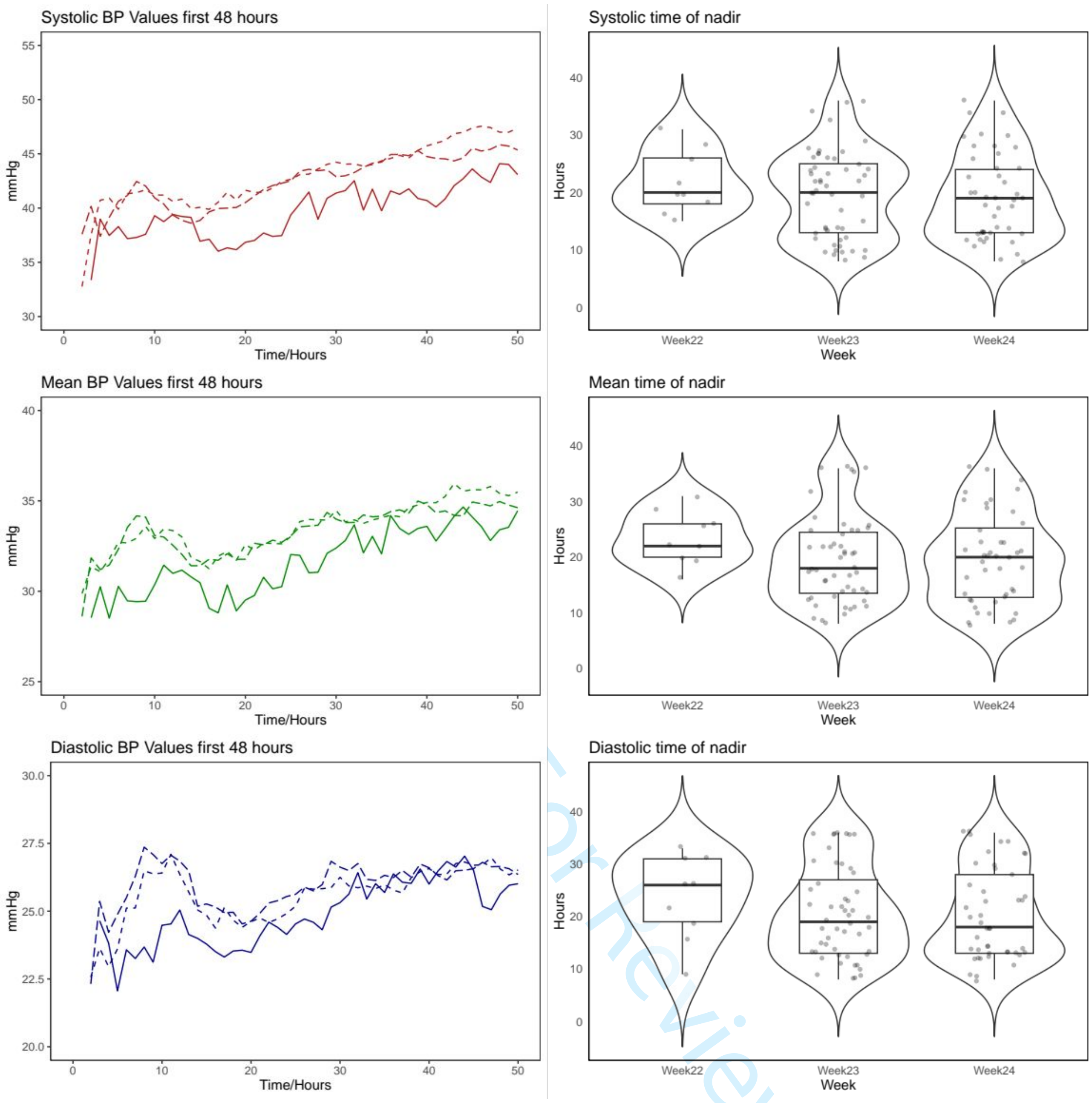

Supplementary Figure 4. Average blood pressure values (left panel column) and time of nadir-point as determined on a per-patient basis during hours 8-36 (right panel column), illustrating the difference by GA week. Top row corresponds to systolic, middle row to mean, and bottom row to diastolic blood pressure. For left column, solid line corresponds to week 22, long-dashed line to week 23 and short-dashed line to week 24.

For Review Only

Tables

Supplementary Table 1. Granger test statistics for comparison of various conditions. Significant test statistics (in both directions) in bold. Tests were performed with frequency=24 (i.e., one day) and lag=6.

Test	Parameter	F 1vs2	P-value 1vs2	F 2vs1	P-value 2vs1
DopavsNoDopa	S	1.4242	0.2106	1.5966	0.1537
	M	0.7565	0.6054	1.098	0.3674
	**D**	2.762	**0.0149** *	5.4865	**0.00004617** ***
Expired Vs Survived	S	0.7114	0.6411	1.7362	0.1181
	M	1.0648	0.3874	1.2985	0.2628
	D	1.0166	0.4177	2.7913	**0.01401** *
IVH vs NoIVH	S	0.672	0.6725	1.3972	0.221
	M	1.8902	0.08768 .	0.7394	0.6189
	D	1.8553	0.09386 .	0.838	0.5429
NEC Vs NoNEC	*S*	1.0188	0.4163	2.2885	0.03959 *
	**M**	2.1973	**0.04762** *	1.4941	0.1856
	D	2.2921	**0.0393** *	4.1012	**0.0008705** ***
NEC And IVH	S	1.6417	0.1412	1.4911	0.1866
	M	1.1992	0.3112	1.1367	0.3451
	**D**	3.7469	**0.001853** **	2.7138	**0.01647** *
Week22 Vs Week 23	S	2.1184	0.05588 .	0.3234	0.9237
	M	3.7312	**0.001924** **	1.7925	0.1061
	D	1.0904	0.372	2.2688	**0.04126** *
Week22 Vs Week 24	S	1.598	0.1533	**1.4297**	0.2086
	M	0.6603	0.6818	1.1013	0.3656
	D	1.2781	0.2723	0.8565	0.5291
Week23Vs Week 24	S	6.5905	**4.651e-06** ***	**1.7821**	0.1081
	M	2.6125	**0.02034** *	2.13	0.05452 .
	D	4.5404	**0.0003415** ***	2.0958	0.05839 .
Csec Vs Natural	S	3.9531	**0.001194** **	2.2704	**0.04107** *
	M	2.6132	**0.02031** *	1.765	0.1117
	D	1.5071	0.1813	2.1612	0.05121 .
ChorioA Vs NoChorioA	S	3.2583	0.005236 **	1.0051	0.4252
	M	0.8812	0.5109	1.8826	0.08899 .
	D	2.337	0.03586 *	1.1103	0.3602

Confidential: For Review Only

Supplementary Table 2. Output summary of generalized linear model for conditions and analyzed numerical variables. Model: IVH Grade ~ Week + Inotrope/vasoactive drug + NEC + >46mmHg at any time + %Time spent at>46mmHg + Nr.Fluctuations. Data were scaled and centered prior to analysis.

Condition	Estimate	Std. Error	z value	Pr(>|z|)	Sig Level
Week	-0.19562	0.09005	-2.172	0.029833	*
DopaTRUE	0.10363	0.20467	0.506	0.612624	
NECTRUE	-0.21959	0.28044	-0.783	0.433605	
GT46mmHgTRUE	0.59868	0.26861	2.229	0.025825	*
PercGT46mmHg	-0.18566	0.1585	-1.171	0.24146	
NrFluc168	-0.34582	0.09755	-3.545	0.000393	***

Supplementary Table 3. Outcome of Fisher's exact tests for patient outcome. Significant test statistics in bold, weakly significant test statistics in bold italics.

Test of proportions: Condition	Fisher's exact p-value	Odds Ratio	Sig. Diff.
Expired patients on dopa Vs. not on Dopa	1	0.9379379	No
Expired patients on dopa Vs. not on Dopa – IVH diagnosis	0.7569	0.7665124	No
Expired patients on dopa Vs. not on Dopa – NEC diagnosis	0.657	0.5290084	No
Expired patients on dopa Vs. not on Dopa – IVH&NEC diagnosis	0.4697	Inf	No
Patients on Dopa with later diagnosis Vs. Patients not on Dopa with later diagnosis	**0.003197**	**0.3555661**	Yes
Patients on Dopa with later IVH Diagnosis Vs. Patients not on dopa with IVH diagnosis	0.2703	0.6567996	No
Expired patients with IVH diagnosis Vs. No diagnosis	***0.0616***	***0.428422***	No
Expired patients with NEC diagnosis Vs. No diagnosis	**0.02968**	**0.3014454**	Yes
Expired patients with IVH&NEC diagnosis Vs. No diagnosis	**0.01159**	**6.036246**	Yes
IVH Diagnosis GA week 22 vs week 23	0.1812	0.2950553	No
IVH Diagnosis GA week 22 vs week 24	0.4857	0.4885168	No
IVH Diagnosis GA week 23 vs week 24	0.2462	1.656861	No
Patient Exceeded/Did not exceed 46mmHg and Expired	0.2567	1.735024	No
Patient Exceeded/Did not exceed 46mmHg and diagnosed with IVH	0.1698	1.833681	No
IVH Diagnosis Csec vs Norm	0.3632	0.6829341	No
IVH Diagnosis ChoriaA diagnosis vs No diagnosis	0.06121	0.4342773	No

Supplementary Table 4. T-test statistics for comparison of blood pressure values at various time-points and conditions.

S/Hours	Week22/Week23	Week22/Week24	Week23/Week24	Expired/Survived	Dopa/NoDopa	IVH/NoIVH	NEC/NoNEC
**3**	t = -3.6317, df = 5.8753, p-value = 0.01135	NS	NS	NS	NS	NS	NS
**12**	NS	NS	NS	NS	NS	NS	NS
**24**	t = -2.8478, df = 11.14, p-value = 0.01568	t = -2.7703, df = 12.447, p-value = 0.01646	NS	NS	NS	NS	NS
**36**	NS	NS	NS	NS	t = -2.0215, df = 90.167, p-value = 0.0462	NS	t = -2.4728, df = 34.845, p-value = 0.01843

Confidential: For Review Only

**48**	NS	NS	NS	NS	t = -2.5818, df = 86.004, p-value = 0.01152	NS	NS
**M/Hours**	**Week22/Week23**	**Week22/Week24**	**Week23/Week24**	**Expired/Survived**	**Dopa/NoDopa**	**IVH/NoIVH**	**NEC/NoNEC**
**3**	t = -2.7417, df = 12.138, p-value = 0.01771	NS	NS	NS	NS	NS	NS
**12**	NS	NS	NS	NS	NS	NS	NS
**24**	NS	NS	NS	NS	NS	NS	NS
**36**	NS	NS	NS	NS	t = -2.0005, df = 103.67, p-value = 0.04806	NS	t = -2.4792, df = 35.259, p-value = 0.01809
**48**	NS	NS	NS	NS	NS	NS	NS
**D/Hours**	**Week22/Week23**	**Week22/Week24**	**Week23/Week24**	**Expired/Survived**	**Dopa/NoDopa**	**IVH/NoIVH**	**NEC/NoNEC**
**3**	NS	NS	NS	NS	NS	NS	NS
**12**	NS	NS	NS	NS	NS	NS	NS
**24**	NS	NS	NS	NS	NS	NS	NS
**36**	NS	NS	NS	NS	NS	NS	NS
**48**	NS	NS	NS	NS	NS	NS	NS

Description of data extraction and statistical analyses

Data extraction, normalization, and removal of outliers

The output from the data monitoring systems in Microsoft Excel format were converted to TSV files and an in-house developed perl-script was used to average the values (recorded on a nominal one datapoint per minute but displaying gaps in the data) on a per-hour basis in order to compensate for gaps in the data. Furthermore, time elapsed since birth to beginning of monitoring was taken into account, resulting in normalized data comparable between patients.

Due to rapidly decreasing amount of data after the 168-hour mark (corresponding to approximately one week after birth), a cut-of at this hour was implemented and no later data-points were included in the analysis.

In order to account for spurious data, caused by measurement errors and other factors unlikely to have a biological/physiological basis, interquartile range criterion (IQR) was used to clean the data and remove outliers. This resulted in removal of 299, 602 and 535 datapoints for the systolic, mean and diastolic datasets, respectively.

Granger's causality tests for time-series data

Granger causality test is a statistical method used to determine whether one time series is a useful predictor of another time series. In other words, it can determine if a change in one variable can be used to predict the change in another variable. In the analysis presented here, it was used to determine if the BP curves were statistically similar enough that one curve could be used to predict another for a given set of conditions, i.e., if statistical similarity exist between BP development (e.g., inotrope/vasoactive drug having been given or not). If one curve is not able to predict the development of another, we can conclude that they are statistically significantly different, given that this is significant in both directions.

Confidential: For Review Only

To account for the fact that the data was analysed in aggregate, i.e., the mean of all patients exhibiting a specific set of conditions, and that patients are likely to display variation in individual development, a frequency of 24 hours and a lag of 6 hours was used in the comparisons.

The "lmtest (version 0.9-40)" R package was utilized for all time-series analysis.

Effect of BP in specific "bands," and identification of dangerous BP thresholds

The procedure described in Vesoulis *et al.* was repeated for the time each patient spent in specific "bands," corresponding to percent of total measurement time spent between specific defined thresholds of blood pressure.

However, no correlation between outcome (e.g., grade of IVH, patient expired) and percentage spent at specific BP values could be found in our data. However, a statistically significant difference was detected regarding if the patient at any time had exceeded the threshold identified by Vesoulis *et al.,* corresponding to 46mmHg for mean blood pressure.

Further, an attempt was made at identifying alternative thresholds with greater statistical effect than the threshold identified by Vesoulis *et al.* by receiver operating characteristic (ROC) analysis, but no threshold value with greater power could be detected.

Analysis of number of fluctuations in blood pressure, i.e., stableness

To analyse the number of fluctuations/how stable the blood pressure was for each patient, an analysis was performed where all patients with a maximum of half the time-points missing, and the missing time-points fixed by linear interpolation with the R na.interp function were included in the analysis. Subsequently, the number of times each patient blood pressure crossed its own 24-hour rolling mean was counted. This concept is illustrated for a singular patient in supplementary Figure 4, where the red line corresponds to the rolling mean and the black line corresponds to the actual BP values.

Confidential: For Review Only

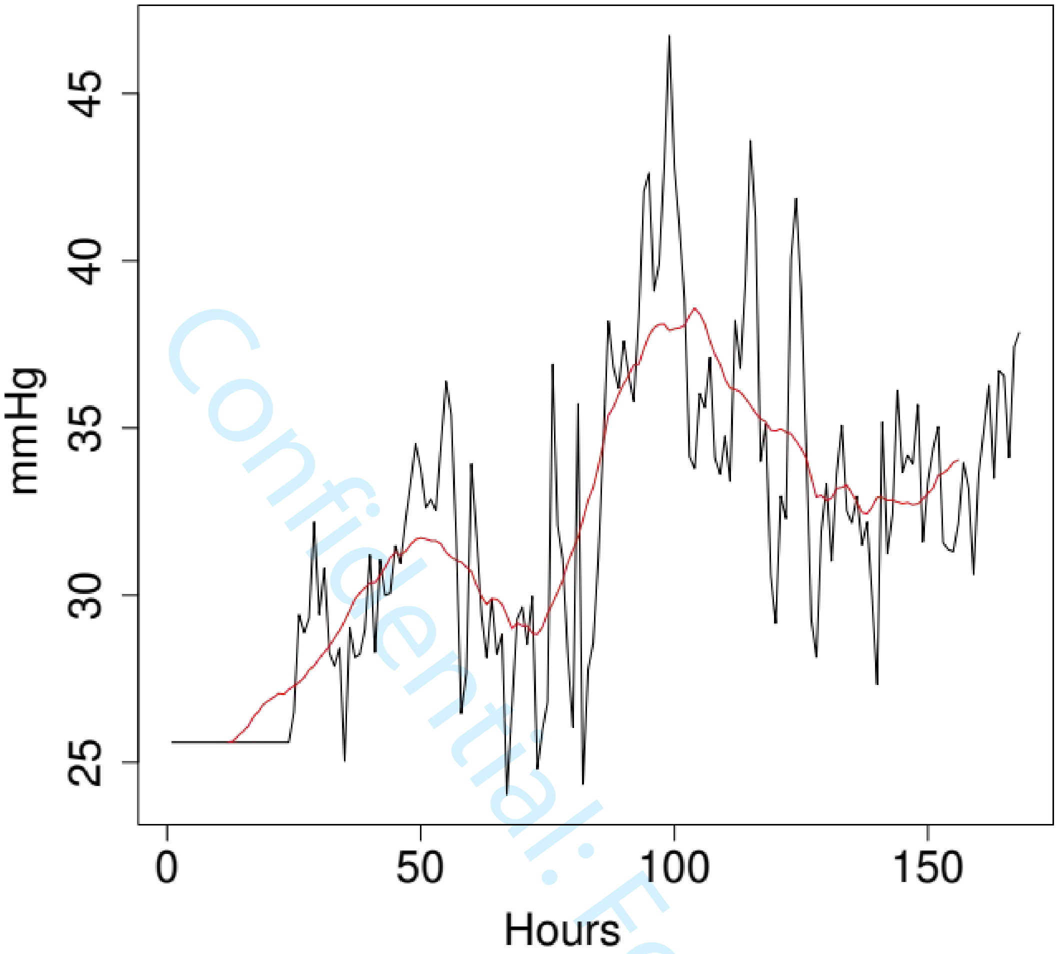

Supplementary Figure 4. Illustration of concept of analysis of number of fluctuations.

In other words, a numerical value was obtained for each patient measuring how many fluctuations took place around the rolling mean, giving a measurement indicating the how stable blood pressure is/slow response to divergent blood pressure.

Determination of nadir-point for individual patients

To detect the nadir point for individual patients, data-series with a maximum of 25% of the data-points missing were selected, and the R function cpt.mean from the "changepoint (version 2.2.4)" R package was used after linear interpolation with the R na.interp function to fix missing datapoints. In order to avoid spurious detections, the analysis was limited to the period between 8 and 36 hours. The parameters for cpt.mean were set as follows: penalty="MBIC",pen.value=0,method="AMOC",Q=5,test.stat="Normal",class=FALSE, param.estimates=TRUE,minseglen=1.

Evaluation of significant factors for inclusion in modelling

In order to evaluate potential factors to include in the general linearized model, a number of different models were evaluated, leading to the final model incorporating factors either previously described as being significant in the literature, or where a Fisher's exact test for our own data indicated that the outcome was numerically different, or where other analysis had suggested that explanatory power was present as in the case of number of fluctuations. A presentation of the factors evaluated can be found in Supplementary table 3. Akaike Information Criterion (AIC) was used to select the final

Confidential: For Review Only

model providing the best fit. The final model corresponded to: IVH Grade ~ Week + Inotrope/vasoactive drug + NEC + >46mmHg at any time + %Time spent at>46mmHg + Nr.Fluctuations.

Confidential: For Review Only

BMJ Paediatrics Open

Blood pressure trends following birth in infants born under 25 weeks' gestational age: a retrospective cohort study

Journal:	*BMJ Paediatrics Open*
Manuscript ID	bmjpo-2023-002438.R1
Article Type:	Original research
Date Submitted by the Author:	14-Feb-2024

Complete List of Authors:	Persad, Emma; Karolinska Institutet, Department of Women's and Children's Health; Karolinska University Hospital, Department of Neonatology<br>Brindefalk, Björn; Stockholm University, Department of Molecular Biosciences, The Wenner-Gren Institute<br>Rakow, Alexander; Karolinska Institutet, Department of Women's and Children's Health; Karolinska University Hospital, Department of Neonatology

Keywords:	Cardiology, Epidemiology, Neonatology

SCHOLARONE™
Manuscripts

BMJ

I, the Submitting Author has the right to grant and does grant on behalf of all authors of the Work (as defined in the below author licence), an exclusive licence and/or a non-exclusive licence for contributions from authors who are: i) UK Crown employees; ii) where BMJ has agreed a CC-BY licence shall apply, and/or iii) in accordance with the terms applicable for US Federal Government officers or employees acting as part of their official duties; on a worldwide, perpetual, irrevocable, royalty-free basis to BMJ Publishing Group Ltd ("BMJ") its licensees and where the relevant Journal is co-owned by BMJ to the co-owners of the Journal, to publish the Work in this journal and any other BMJ products and to exploit all rights, as set out in our licence.

The Submitting Author accepts and understands that any supply made under these terms is made by BMJ to the Submitting Author unless you are acting as an employee on behalf of your employer or a postgraduate student of an affiliated institution which is paying any applicable article publishing charge ("APC") for Open Access articles. Where the Submitting Author wishes to make the Work available on an Open Access basis (and intends to pay the relevant APC), the terms of reuse of such Open Access shall be governed by a Creative Commons licence – details of these licences and which Creative Commons licence will apply to this Work are set out in our licence referred to above.

Other than as permitted in any relevant BMJ Author's Self Archiving Policies, I confirm this Work has not been accepted for publication elsewhere, is not being considered for publication elsewhere and does not duplicate material already published. I confirm all authors consent to publication of this Work and authorise the granting of this licence.

Confidential: For Review Only

Confidential: For Review Only

Cover page

Full title

Blood pressure trends following birth in infants born under 25 weeks' gestational age: a retrospective cohort study

Authors

Emma Persad[1,2]

Björn Brindefalk[3]

Alexander Rakow[1,2*]

Authors' institutional affiliations

[1]Department of Women's and Children's Health, Karolinska Institutet, Solna, Sweden

[2]Department of Neonatology, Karolinska University Hospital, Solna, Sweden

[3]Department of Molecular Biosciences, The Wenner-Gren Institute, Stockholm University, Stockholm, Sweden

Corresponding author

Alexander Rakow, Department of Women's and Children's Health, Karolinska Institutet, Tomtebodavägen 18A, 17177 Solna, Sweden, alexander.rakow@ki.se, +46725968444

Abstract

Objective

The aim of our study was to describe post-natal blood pressure (BP) trends and evaluate relevant dynamics and outcomes for a subgroup of extremely preterm (EPT) infants.

Design

Retrospective observational cohort study.

Setting

Patients admitted to Karolinska University Hospital Stockholm.

Patients

EPT infants born between 22+0 and 24+6 weeks' gestational age (GA) undergoing invasive, continuous BP monitoring through an umbilical arterial catheter.

Main outcome measures

Physiological BP trends, the influence of cardiovascular active interventions and fluid boluses on BP, and relevant adverse outcomes, including intraventricular hemorrhage (IVH), necrotizing enterocolitis (NEC), and death were mapped over the first week of life.

Results

We included 125 infants between January 2009 and November 2021. Mean BP values were 31 mmHg, 32 mmHg, and 35 mmHg, at 3 hours, 24 hours, and 48 hours, respectively. A pronounced BP dip and nadir was observed around 20 hours, with a mean BP value of 32 mmHg. Eighty-four percent received fluid boluses within the first week of life, however we could not observe any noteworthy change in BP following administration. Only 8% of patients received cardiovascular active drugs, which were too few to infer drug-specific effects. Overall, 48% developed IVH, 15% developed NEC, and 25% died.

Conclusions

Approximating clinically acceptable mean BP values using GA gives under-estimations in these infants. The post-natal BP dip should be regarded as a physiological phenomenon and not automatic grounds for interventions which may momentarily stabilize BP but have no appreciable short- or long-term effects. Further studies are warranted for improved understanding of clinically relevant trends and outcomes.

Confidential: For Review Only

What is already known on this topic

Low blood pressure (BP) affects 15-50% of extremely preterm (EPT) infants following birth and is associated with adverse outcomes, however, high fidelity granular data outlining acceptable BP trends and accurate BP-estimation practices crucial for comprehensive patient monitoring are lacking in this cohort. Further, a post-natal BP dip has been observed in EPT infants although is not yet well-described or widely acknowledged, warranting clarification on the magnitude and timing of this dip and a high-quality descriptive analysis of BP to guide clinical care.

What this study adds

We provide valuable insight into BP trends observed in a typical group of 125 EPT infants born at the border of viability (22+0 to 24+6 weeks' gestational age [GA]), finding a significant drop in BP and a nadir reached at approximately 20 hours after birth, regardless of cardiovascular active drug and fluid bolus administration. Additionally, we show that methods for predicting BP based on GA significantly underestimate the actual BP levels in this cohort.

How this study might affect research, practice or policy

Clinical guidelines and practitioners should recognize the post-natal BP dip as a physiological occurrence, avoid GA-based BP estimation practices, acknowledge daily increments of BP, and strive to utilize additional indicators of perfusion to access hemodynamic stability during assumed hypotensive episodes.

Confidential: For Review Only

INTRODUCTION

Despite increasing ability to care for extremely preterm infants (EPT), defined as those born under 28 weeks' gestational age (GA), considerable uncertainty surrounding age- and condition-appropriate blood pressure (BP) remains (1-6). Furthermore, data on the efficacy of anti-hypotensive treatment and relevant thresholds to initiate treatment are lacking, despite episodes of assumed hypotension often routinely being targeted with cardiovascular active drugs or volume boluses potentially associated with increased morbidity (7, 8). Comparatively, not intervening during hypotensive episodes can be equally harmful, particularly for neurodevelopment (9-13). Clinical management of these infants is further complicated by their rapidly-evolving physiology and numerous medical interventions, which may influence or mask natural BP trends (14).

Neonatal intensive care units (NICUs) care for an increasing number of EPT infants prone to severe outcomes and complications, such as intraventricular hemorrhage (IVH), necrotizing enterocolitis (NEC), and early neonatal death (4, 5). Although BP values for infants aged 23 to 25 weeks' GA have been reported in the literature, there are limited high fidelity granular BP data on these infants, particularly as very few centers have access to comprehensive databases and invasively-measured continuous monitoring data (13).

A post-natal BP dip and immaturity-related BP fluctuations have been observed in EPT infants, although a comprehensive exploration of these phenomena and their long-term implications are not well-described (3, 15). Despite these BP variations being reported in the literature, clinicians commonly follow the consensus-based practice of estimating acceptable mean BP using the infants' GA in weeks (13, 16, 17). Hypotension in EPT infants has also been defined as mean arterial pressure <30 mmHg or less than the 10th percentile of gestational and postnatal age from published norms, which are often based on non-invasive measurements prone to over-estimations

Confidential: For Review Only

and artifacts (2, 3, 18). As providing evidence-based, high-quality care is crucial for these vulnerable infants and assumed hypotensive episodes often prompt invasive interventions to stabilize BP, clarification on age-appropriate BP and the magnitude and timing of the post-natal dip is warranted for improved clinical management. This study aims to describe BP in EPT infants born before 25 weeks' GA in our single-center cohort, providing insight into the physiological development and outcomes of a typical group of vulnerable, underdeveloped patients subject to multiple supportive interventions.

METHODS

This was a retrospective observational cohort study in EPT infants admitted to Karolinska University Hospital Stockholm between January 2009 and November 2021. We included all infants born between 22+0 and 24+6 weeks' GA with an umbilical arterial catheter enabling continuous invasive BP monitoring recorded at one-minute intervals. Infants over 25+0 weeks' GA, without invasive monitoring, and with chromosomal abnormalities were excluded (Supplementary Figure 1).

Patient Monitoring Solutions (Clinisoft GE®) was used to identify eligible patients using both GA and Diagnosis Related Group codes and retrieve monitoring data for all patients to limit selection bias. Patient documentation systems (TakeCare® and Obstetrix®) were used to extract demographic information and outcomes. All non-anonymized data were stored on Region Stockholm servers and not shared externally.

Data were extracted for BP values (systolic, mean, diastolic), time between birth and monitoring, incidence and time of IVH, NEC, and death, and demographic factors, including GA (weeks + days), time of birth, weight (grams), vasopressor or inotropic drugs (dopamine and dobutamine in this cohort), fluid boluses (fresh frozen plasma and NaCl), respiratory support, mode of delivery,

Confidential: For Review Only

maternal steroids, and maternal chorioamnionitis. Our institution follows a protocolized treatment pathway for the provision of hypotension medication (19).

We normalized the time elapsed from birth until start of invasive measurement, aggregating data on a per-hour basis to account for un-even sampling (in-house Perl script available on request). We focused our investigation between hours 1 and 168 of life, corresponding to a maximum of 76 million per-minute measurements, resulting in 16555, 16390, and 16354 data points following interquartile range-based outlier removal for systolic, mean, and diastolic BP values, respectively (Supplementary Figure 2).

All statistical testing and data-handling apart from initial extraction was done with R Statistical Software (version 4.0.3) (20). We evaluated statistically significant differences in time-series using Granger's causality tests with bidirectional significance indicating similarity between curves. Modelling of significant factors for IVH severity was done with a generalized linear model (GLM) where numerical variables were centered and re-scaled and other variables treated as factors (Model: IVH Grade ~ Week + Inotrope/vasoactive drug + NEC + >46mmHg at any time + %Time spent at>46mmHg + Nr.Fluctuations). For further analysis details, see supplementary material. We did not involve patients or the public in the design, conduct, reporting, or dissemination plans.

RESULTS

Demographics

This study included 125 infants born below 25 weeks' GA at Karolinska University Hospital Stockholm between 2009 and 2021. Our cohort comprised 11, 60, and 54 infants born at week 22, 23, and 24, respectively. Table 1 outlines baseline demographic data.

Table 1.

Confidential: For Review Only

Blood pressure trends during the first 48 hours

Increasing BP trends were observed over time. Our cohort had a mean BP of 31 mmHg at baseline (3 hours after birth), 32 mmHg at 24 hours (1 mmHg increase from baseline), and 35 mmHg at 48 hours (4 mmHg increase from baseline). The BP nadir occurred at approximately 20 hours, with a mean BP of 32 mmHg. Average systolic, mean, and diastolic BP values and incremental changes from baseline are presented in Table 2.

Table 2.

Figure 1a illustrates mean BP trends for all infants with the 25th and 75th quartiles, showing an initial increase and subsequent decrease during the first 24 hours. Figure 1b presents simplified findings suitable for clinical use.

Figure 1.

Figure 2 illustrates BP trends for all infants during the first 48 hours with a pronounced dip and nadir observed at approximately 20 hours following birth.

Figure 2.

Figure 3 illustrates the timing of the nadir for systolic, mean, and diastolic BP values grouped by GA week.

Figure 3.

Differences in blood pressure trends for various conditions

Granger's continuity tests showed significantly different BP trends when considering aggregate data for most conditions (Supplementary Table 1). A refined representation of BP trends for infants in the three GA cohorts and plots comparing infants that died vs. survived and developed IVH or

Confidential: For Review Only

NEC vs. did not during the first 168 hours of life are shown in Figure 4 (Figure 4; Supplementary Table 3).

Figure 4.

Model analysis and correlation of various factors to IVH

Using a GLM, we tested time spent in mean BP-bands >46 mmHg or <23 mmHg to evaluate potential correlations with IVH grade as reported by Vessoulis *et al*, however found no such correlations (Supplementary Table 2).(15) Interestingly, we found a significant correlation between infants exceeding a mean BP of 46 mmHg at least once and the appearance of IVH at higher grades (p=0.02). To evaluate BP fluctuations, we calculated the number of the times BP crossed its own 24-hour moving average for a numerical measurement of BP stability, finding that a slow response to divergent BP was strongly correlated to the appearance of IVH at higher grades (p≤0.001). We also observed a statistically significant correlation between severity of IVH grade and lower GA (p=0.03) (Supplementary Figure 4).

DISCUSSION

This study provides high fidelity granular BP data in infants born between 22 and 25 weeks' GA, contributing further insight to a limited body of evidence on this extremely vulnerable population (3). Between 2009 and 2021, the clinical management of EPT infants at our center, comprising ventilation strategies, fluid and bolus management, inotropic use, and tolerance of hypercapnia, saw minimal changes. This stability can be attributed to our center's conservative approach, emphasizing the use of established and reliable methodologies. Notably, our ventilation strategy has remained unchanged since 2003, and the array of cardiovascular active drugs administered has not altered significantly. The only noteworthy modifications include the admission of infants born

Confidential: For Review Only

at 22 weeks' GA from 2016 and an increased emphasis on delayed cord clamping from 2018, meaning the overall management framework has adhered to a rigid, time-tested paradigm. As interventions to address suspected hypotensive BP can vary across countries and medical centers, our single-center perspective provides a clear and consistent snapshot of our institution's practices and enhances the robustness of our findings.

Our cohort illustrated non-linear BP dynamics, finding a decrease in BP following birth with a nadir at approximately 20 hours (Figure 2; Figure 3). We also observed significant differences in BP trends when comparing infants born at 22, 23, or 24 weeks' GA, highlighting the potential effect of immaturity on physiology, even for only a 1-2 week difference.

Despite minimal GA differences between our cohorts, Batton *et al*. described a considerably earlier decrease in BP, noting a nadir at only 4-6 hours following birth (3). The transition from invasive to non-invasive measurement techniques was proposed to partly account for the observed decline in BP (3). As our cohort exclusively received invasive BP measurements, we consider the timing and dip in BP to be seemingly physiological and not attributable to measurement error, which is of immense clinical relevance as it often coincides with the initiation of anti-hypotensive treatment. Although the physiological mechanisms behind the dip remain elusive, Banerjee *et al*. have suggested that a reduction in cardiac output may be a potential explanation (21). Interestingly, they demonstrate a decrease in cardiac output with a nadir at approximately 12 hours primarily driven by a decrease in heart rate, which considerably precedes the BP drop seen in our cohort at 24 hours (Supplementary Figure 3). We further investigated the relationship between heart rate and BP development, observing that BP began to recover approximately 10 hours before there was a corresponding increase in heart rate (Supplementary Figure 3). Cardiac output has previously been shown to correlate poorly with mean BP, suggesting that other factors, such as an increase in

Confidential: For Review Only

left-to-right shunt through a patent ductus arteriosus or changes in mean airway pressure due to the progression of respiratory distress syndrome (HFOV), should also be considered (22, 23). Further research into neuroendocrine factors, including catecholamines and hormones affecting systemic and pulmonary vascular resistance, should be prioritized to uncover the mechanisms underlying postnatal physiological development (24).

Our findings add to the literature challenging the practice of GA-based estimation of post-natal BP in EPT infants (15). In our cohort, infants born between 22+0 and 24+6 weeks' GA had a mean BP of 31 mmHg at 3 hours, 32 mmHg at 24 hours, and 35 mmHg at 48 hours, considerably higher than estimated using GA-based practices. Our findings were similar to Vesoulis *et al.*, who estimated mean BP in infants <28 weeks' GA as 33 mmHg following birth (15). Similarly, Kent *et al*. reported a mean BP range of 31-43 mmHg at 24 hours in infants aged 28-29 weeks' GA (18). This encompasses our findings of 32 mmHg, although their measurements were recorded non-invasively, potentially explaining their high upper range of 43 mmHg. Similarly, Batton *et al.* reported a BP increase of 0.2 mmHg per hour, an increase which our cohort did not exhibit; however, they did not strictly use invasive measurement techniques, which remain the most reliable means of BP monitoring (3).

Different mean BP trends were found between major comparisons, indicating that eventual death, IVH diagnosis alone, IVH and NEC diagnosis combined, GA, maternal factors, and delivery method may be correlated to mean BP trends or influence mean BP enough to alter trends when compared to infants in the respective comparison groups (Supplementary Table 1). However, these tests only revealed differences in trends and not that one trend could be connected to specific

Confidential: For Review Only

outcomes, and the findings were only evident when considering aggregate data. Further exploration is required to draw more in-depth conclusions.

We found no correlation between time spent in specific mean BP-bands suggested as thresholds (>46 mmHg and <23 mmHg) and eventual IVH diagnosis (15). However, infants who had a mean BP that crossed 46 mmHg at any time were significantly correlated to more severe IVH grade. Further, our data did not show the same correlation between increased instability and increased severity of IVH observed by Vesoulis *et al*., but instead found that a slow response to divergent BP was strongly correlated to increased IVH severity. However, cohort differences and Vesoulis *et al*. not detailing their statistical analysis rendered a direct comparison difficult.

While guidelines outlining thresholds for intervention would be clinically useful, our analyses highlight that the complexity of BP trends renders numerical values difficult to generalize and utilize. Retrospectively analyzing BP trends over time may display patterns indicating a risk for adverse outcomes, however, apart from having crossed a mean BP of 46 mmHg, we could not identify stringent numerical limits to define clinically safe BP thresholds. Although clinicians are particularly inclined to intervene to stabilize BP in EPT infants, our data showed a seemingly natural dip following birth and no correlation between low BP and adverse outcomes (15, 18). If regarded as a natural physiological reaction, this dip should not be automatic grounds for systemic intervention, which may even be counter-productive considering the potential risks. Thus, it is inadequate to rely solely on BP to assess hemodynamic stability in clinical practice and imperative to utilize additional indicators of perfusion before commencing interventions.

This study provides quantitative and qualitative improvements to the literature, namely the high fidelity granular data continuous data in a comparatively large infant cohort for this GA group.

Confidential: For Review Only

Our cohort also had a narrow GA range and very few received cardiovascular active drugs, allowing us to infer trends representative of this understudied population. However, our results were limited by only evaluating 11 infants born in week 22. Although this is novel for the literature, the findings for this subset should be viewed with caution. It is unclear whether the lesser fluctuations observed in this group are due to immaturity-related physiological factors or low patient numbers. Further, 93% of infants were on HFOV, as this our first line ventilation strategy for EPT infants, which has the potential to affect preload and afterload and thereby cardiac output, subsequently decreasing BP when high mean airway pressures are used. Only 8% of infants received cardiovascular active drugs during the first week of life, which we considered too few to investigate drug-related effects. Eighty-four percent of our infants received fluid boluses or fresh frozen plasma during the first week of life, however we could not observe any noteworthy change in BP following administration. We were also not able to obtain data on cord clamping procedures. Regardless, as it can be assumed that most infants in this age cohort receive the aforementioned interventions routinely from 2018, these results are still indicative of this population. Nevertheless, these values should be regarded as approximate guidelines when treating infants in this age range.

CONCLUSION

This study presents highly accurate, invasive BP data on a typical cohort of unstable EPT infants born under 25 weeks' GA. Our findings highlight the post-natal BP dip as a likely physiological phenomenon of currently unclear etiology which should not immediately prompt medical intervention provided other indicators of perfusion are favourable. Our results also challenge the GA-based practice of estimating mean BP, encouraging clinicians to reconsider this rule and acknowledge its vast underestimation in these infants. Overall, our data did not reveal specific BP trends to be correlated with poor outcomes, challenging the use of BP as a sole indicator of

For peer review only

hemodynamic stability and warranting future investigations. Further research using invasive data on larger cohorts of EPT infants is necessary to clarify acceptable physiological BP trends to optimize clinical care.

Acknowledgements

We would like to thank Raman Abram for his technical support and guidance with the patient databases.

Funding

This research received no specific grant from any funding agency in the public, commercial or not-for-profit sectors.

Competing interests

The authors have no potential conflicts of interest to report.

Contributors

EP made substantial contributions to the design and acquisition of data, drafted the article and revised it critically for important intellectual content, and approved the final version to be published.

BB made substantial contributions to the analysis and interpretation of data, revised the article critically for important intellectual content, and approved the final version to be published.

Confidential: For Review Only

AR made substantial contributions to the conception and design of the study, interpreted the data, drafted the article and revised it critically for important intellectual content, and approved the final version to be published.

Research Ethics Approval

Ethical approval was obtained from the Swedish Ethical Review Authority (Etikprövningsmyndigheten, approval number: 2022-01588-01-262498).

Patient consent for publication

Not applicable

Data availability statement

The data supporting the findings of this study (deidentified participant data) are available upon reasonable request to the corresponding author.

Confidential: For Review Only

References

1. Kent AL, Meskell S, Falk MC, Shadbolt B. Normative blood pressure data in non-ventilated premature neonates from 28-36 weeks gestation. Pediatr Nephrol. 2009;24(1):141-6.
2. Miall-Allen VM, de Vries LS, Whitelaw AG. Mean arterial blood pressure and neonatal cerebral lesions. Arch Dis Child. 1987;62(10):1068-9.
3. Batton B, Li L, Newman NS, Das A, Watterberg KL, Yoder BA, et al. Evolving blood pressure dynamics for extremely preterm infants. J Perinatol. 2014;34(4):301-5.
4. Development of audit measures and guidelines for good practice in the management of neonatal respiratory distress syndrome. Report of a Joint Working Group of the British Association of Perinatal Medicine and the Research Unit of the Royal College of Physicians. Arch Dis Child. 1992;67(10 Spec No):1221-7.
5. Pejovic B, Peco-Antic A, Marinkovic-Eric J. Blood pressure in non-critically ill preterm and full-term neonates. Pediatr Nephrol. 2007;22(2):249-57.
6. Batton B, Batton D, Riggs T. Blood pressure during the first 7 days in premature infants born at postmenstrual age 23 to 25 weeks. Am J Perinatol. 2007;24(2):107-15.
7. Bakshi S, Koerner T, Knee A, Singh R, Vaidya R. Effect of Fluid Bolus on Clinical Outcomes in Very Low Birth Weight Infants. J Pediatr Pharmacol Ther. 2020;25(5):437-44.
8. Joynt C, Cheung PY. Treating Hypotension in Preterm Neonates With Vasoactive Medications. Front Pediatr. 2018;6:86.
9. Fanaroff AA, Fanaroff JM. Short- and long-term consequences of hypotension in ELBW infants. Semin Perinatol. 2006;30(3):151-5.
10. Verma RP, Dasnadi S, Zhao Y, Chen HH. Complications associated with the current sequential pharmacological management of early postnatal hypotension in extremely premature infants. Proc (Bayl Univ Med Cent). 2019;32(3):355-60.
11. Schwepcke A, Weber FD, Mormanova Z, Cepissak B, Genzel-Boroviczeny O. Microcirculatory mechanisms in postnatal hypotension affecting premature infants. Pediatr Res. 2013;74(2):186-90.
12. Durrmeyer X, Marchand-Martin L, Porcher R, Gascoin G, Roze JC, Storme L, et al. Abstention or intervention for isolated hypotension in the first 3 days of life in extremely preterm infants: association with short-term outcomes in the EPIPAGE 2 cohort study. Arch Dis Child Fetal Neonatal Ed. 2017;102(6):490-6.
13. Faust K, Hartel C, Preuss M, Rabe H, Roll C, Emeis M, et al. Short-term outcome of very-low-birthweight infants with arterial hypotension in the first 24 h of life. Arch Dis Child Fetal Neonatal Ed. 2015;100(5):F388-92.
14. Zubrow AB, Hulman S, Kushner H, Falkner B. Determinants of blood pressure in infants admitted to neonatal intensive care units: a prospective multicenter study. Philadelphia Neonatal Blood Pressure Study Group. J Perinatol. 1995;15(6):470-9.

Confidential: For Review Only

15. Vesoulis ZA, El Ters NM, Wallendorf M, Mathur AM. Empirical estimation of the normative blood pressure in infants <28 weeks gestation using a massive data approach. J Perinatol. 2016;36(4):291-5.
16. van Zadelhoff AC, Poppe JA, Willemsen S, Mauff K, van Weteringen W, Goos TG, et al. Age-dependent changes in arterial blood pressure in neonates during the first week of life: reference values and development of a model. Br J Anaesth. 2023;130(5):585-94.
17. Stranak Z, Semberova J, Barrington K, O'Donnell C, Marlow N, Naulaers G, et al. International survey on diagnosis and management of hypotension in extremely preterm babies. Eur J Pediatr. 2014;173(6):793-8.
18. Kent AL, Kecskes Z, Shadbolt B, Falk MC. Normative blood pressure data in the early neonatal period. Pediatr Nephrol. 2007;22(9):1335-41.
19. Rakow. Nationell riktlinje för neonatala perfusionsproblem och hypotension Svenska Neonatalföreningen; 2021.
20. R. R; A language and environment for statistical computing: R Foundation for Statistical Computing; 2020 [Available from: https://www.R-project.org/.
21. Banerjee J, Khatib N, Mansfield RC, Sathiyamurthy S, Kariholu U, Lees C. Continuous non-invasive measurement of cardiac output in neonatal intensive care using regional impedance cardiography: a prospective observational study. Arch Dis Child Fetal Neonatal Ed. 2023.
22. Kharrat A, Rios DI, Weisz DE, Giesinger RE, Groves A, Yang J, et al. The Relationship between blood pressure parameters and left ventricular output in neonates. J Perinatol. 2019;39(5):619-25.
23. Kluckow M, Evans N. Relationship between blood pressure and cardiac output in preterm infants requiring mechanical ventilation. J Pediatr. 1996;129(4):506-12.
24. Ezaki S, Suzuki K, Kurishima C, Miura M, Moriwaki K, Arakawa H, et al. Levels of catecholamines, arginine vasopressin and atrial natriuretic peptide in hypotensive extremely low birth weight infants in the first 24 hours after birth. Neonatology. 2009;95(3):248-55.

Confidential: For Review Only

Tables

Table 1. Patient demographics, parameters, and outcomes.

**Demographics (125 infants)**	**n /data (%)**
22 weeks' GA	11 (9%)
23 weeks' GA	60 (48%)
24 weeks' GA	54 (43%)
Average weight	627 g
Average time to invasive BP monitoring	222 mins – 3 hrs 42 mins
HFOV received	116 (93%)
Cardiovascular active drugs received within one week	10 (8%)
Fluid bolus received within one week	105 (84%)
IVH any	60 (48%)
IVH Grade I+II / III+IV	45 (75%) / 15 (25%)
NEC	19 (15%)
NEC operated	18/19 (95%)
Death overall	31 (25%)
Death within 2 weeks	13 (10%)
Average age at death overall	35 days
Average age at death if died within 2 weeks	5 days
Vaginal delivery	70 (56%)
Mother given antenatal steroids	108 (86%)
Chorioamnionitis present	31 (25%)

Table 2. Average systolic (S), mean (M), and diastolic (D) blood pressure (mmHg) and incremental change data for all 125 infants over time.

	**Blood pressure (mmHg)**		
**Time**	**S**	**M**	**D**
3 hours	39	31	24
12 hours	40	33	26
24 hours	42	32*	25*

Confidential: For Review Only

36 hours	44	34	26
48 hours	46	35	27
Value at nadir (time)	41 (20 hours)	32 (20 hours)	25 (21 hours)
Increase from 3 hours to 12 hours	1	1	2
Increase from 3 hours to 24 hours	3	1	1*
Increase from 3 hours to 36 hours	6	3	2
Increase from 3 hours to 48 hours	7	4	2
Change between 3 hours and 24 hours	3	1	1
Change between 24 hours and 48 hours	4	3	1

* Indicates a decrease in mmHg between 12 hours and 24 hours

Figures

Figure 1a. Presentation of total data for 125 infants; mean value and quartiles (shaded area) for systolic (red), mean (green), and diastolic (blue) values for hours 1-168. Shading corresponds to first and third quartile for each dataset. **1b.** Plots showing the smoothed quintiles and mean value for systolic, mean, and diastolic data for the first 168 hours, giving a simplified summary of expected BP trends for extremely premature babies. Curves were smoothed with the Loess function and span=0.7 for easier interpretation.

Figure 2. Detailed plot showing BP trends for all infants during the first 48 hours after birth with vertical lines corresponding to the time frames 3-12h, 12-24h, 24-36h and 36-48h). Shading corresponds to first and third quartile for each dataset. The error-bars indicate the mean nadir point and standard deviation for the nadir point for each dataset.

Figure 3. Average blood pressure values (left panel column) and time of nadir-point as determined on a per-patient basis during hours 8-36 (right panel column), illustrating the difference by GA week. Top row corresponds to systolic, middle row to mean, and bottom row to diastolic blood pressure. For left column, solid line corresponds to week 22, long-dashed line to week 23 and short, dashed line to week 24.

Figure 4. Comparison of systolic (red), mean (green), and diastolic (blue) BP trends over time for different week cohorts. Solid lines correspond to week 22, long dashes week 23, and short dashes week 24 GA (left). Solid line indicates base condition (survived, no diagnosis) while dashed line indicates the alternative (right). Curves were smoothed with the Loess function and span=0.1.

Confidential: For Review Only

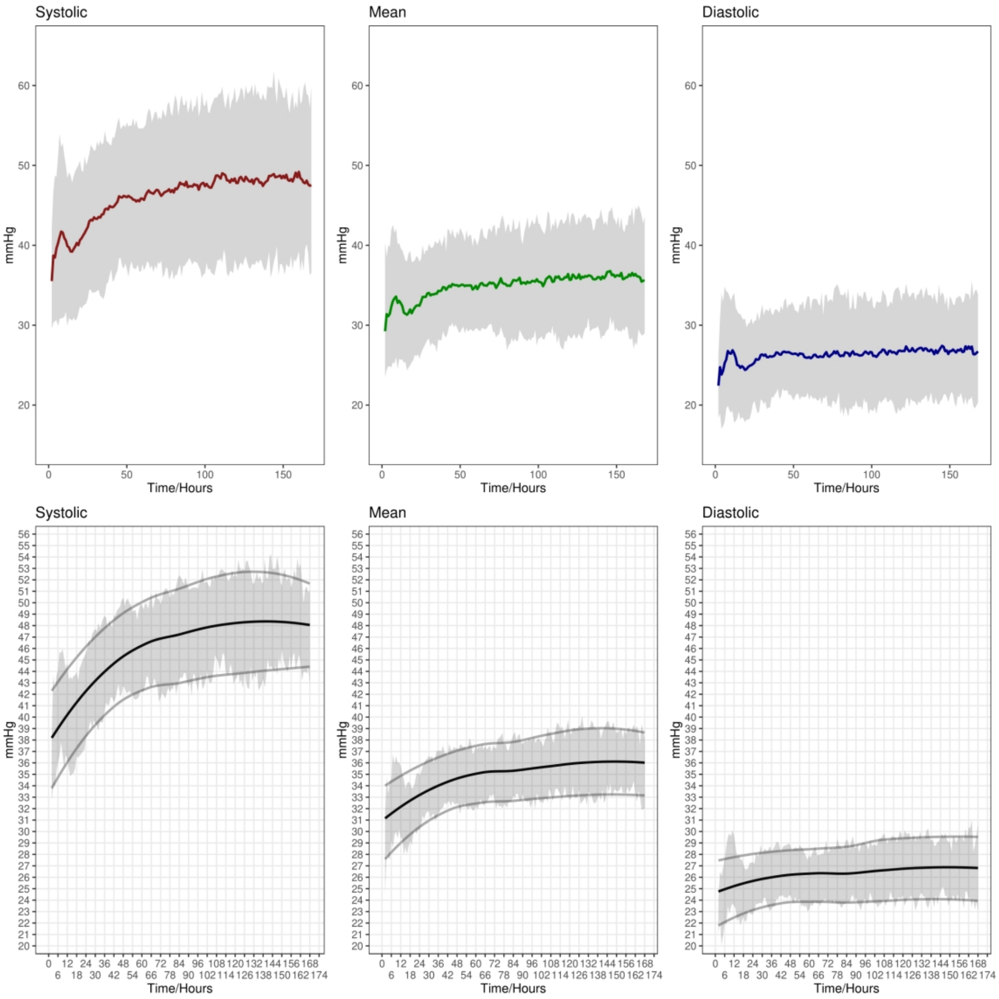

Figure 1. Top: Presentation of total data for 125 infants; mean value and quartiles (shaded area) for systolic (red), mean (green), and diastolic (blue) values for hours 1-168. Shading corresponds to first and third quartile for each dataset. Bottom: Plots showing the smoothed quintiles and mean value for systolic, mean, and diastolic data for the first 168 hours, giving a simplified summary of ex-pected BP trends for extremely premature babies. Curves were smoothed with the Loess function and span=0.7 for easier interpretation.

771x771mm (79 x 79 DPI)

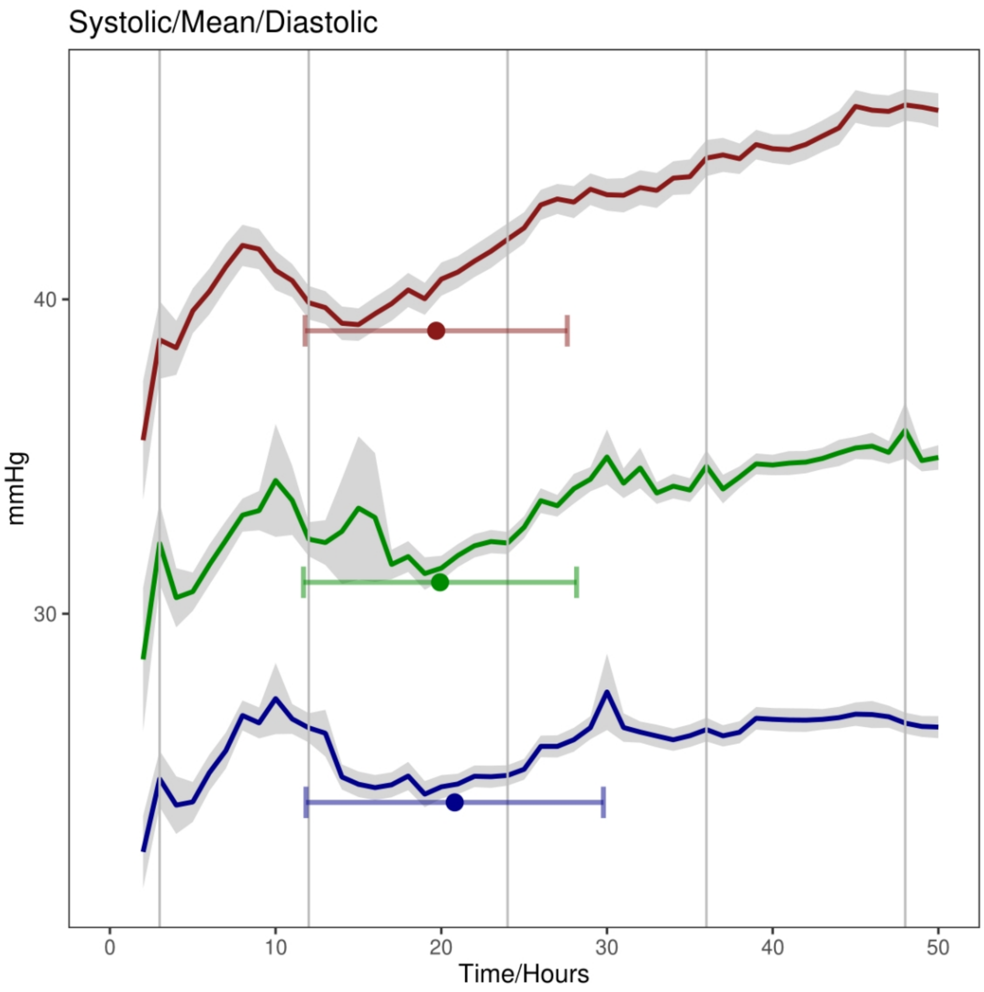

Figure 2. Detailed plot showing BP trends for all infants during the first 48 hours after birth with vertical lines corresponding to the time frames 3-12h, 12-24h, 24-36h and 36-48h). Shading corre-sponds to first and third quartile for each dataset. The error-bars indicate the mean nadir point and standard deviation for the nadir point for each dataset.

385x385mm (79 x 79 DPI)

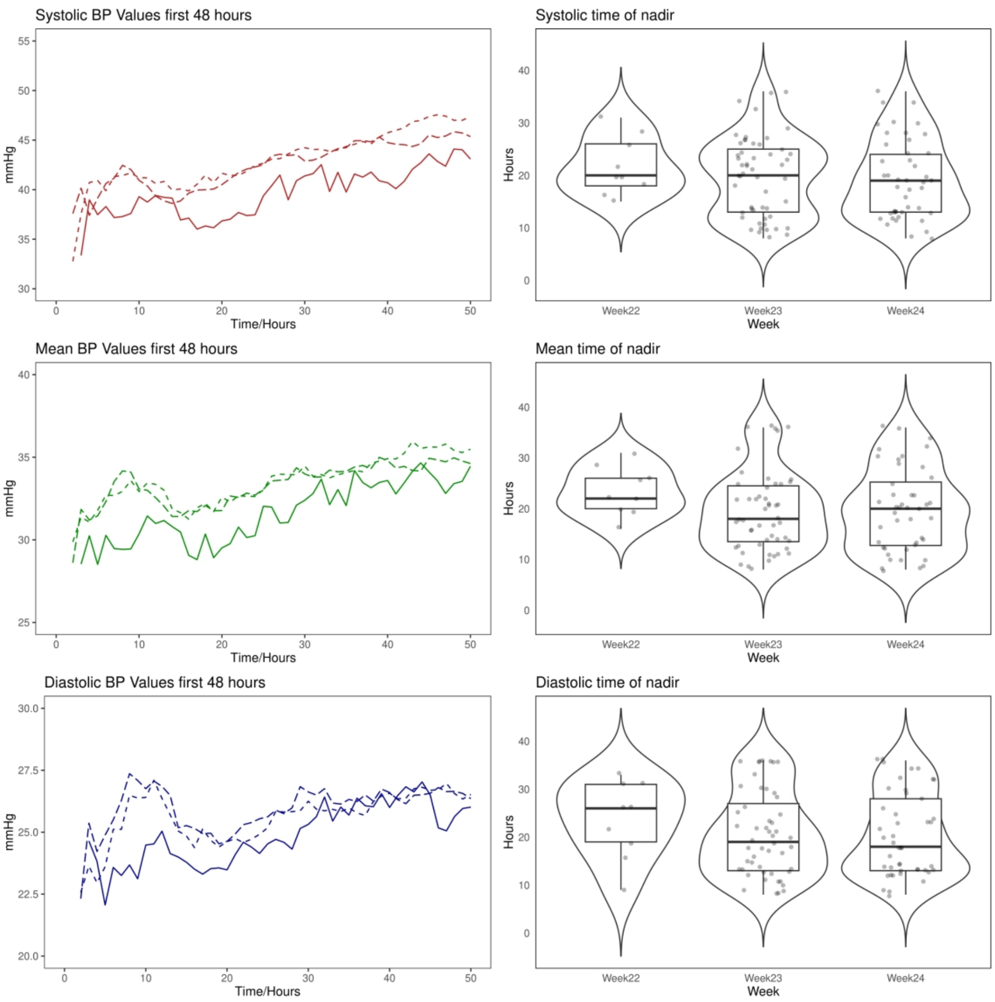

Figure 3. Average blood pressure values (left panel column) and time of nadir-point as determined on a per-patient basis during hours 8-36 (right panel column), illustrating the difference by GA week. Top row corresponds to systolic, middle row to mean, and bottom row to diastolic blood pressure. For left column, solid line corresponds to week 22, long-dashed line to week 23 and short, dashed line to week 24.

771x771mm (79 x 79 DPI)

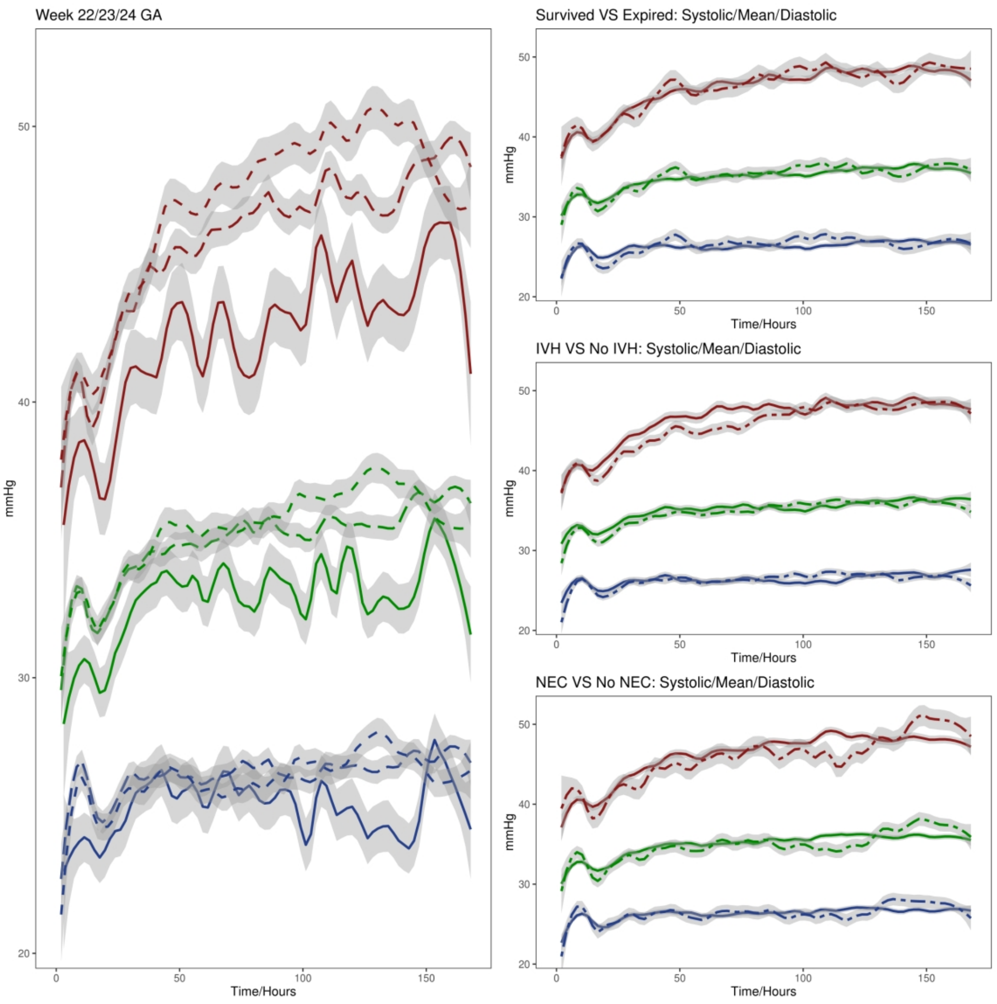

Figure 4. Comparison of systolic (red), mean (green), and diastolic (blue) BP trends over time for different week cohorts. Solid lines correspond to week 22, long dashes week 23, and short dashes week 24 GA (left). Solid line indicates base condition (survived, no diagnosis) while dashed line indicates the alternative (right). Curves were smoothed with the Loess function and span=0.1.

771x771mm (79 x 79 DPI)

Blood pressure trends following birth in infants born under 25 weeks' gestational age - Supplementary Material

Emma Persad, MD, Björn Brindefalk, PhD, Alexander Rakow, MD, PhD

Figures

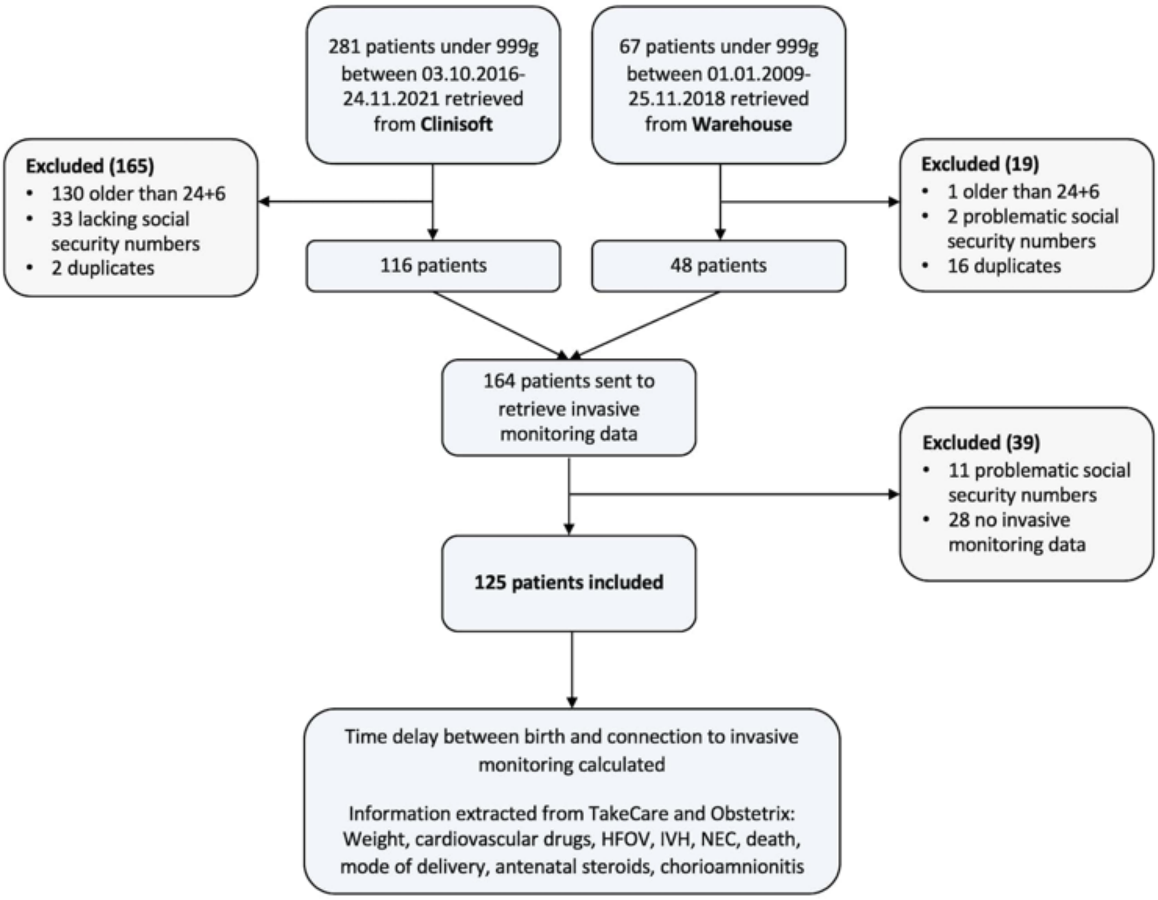

Supplementary Figure 1. Data collection methods and flow of patients into study.

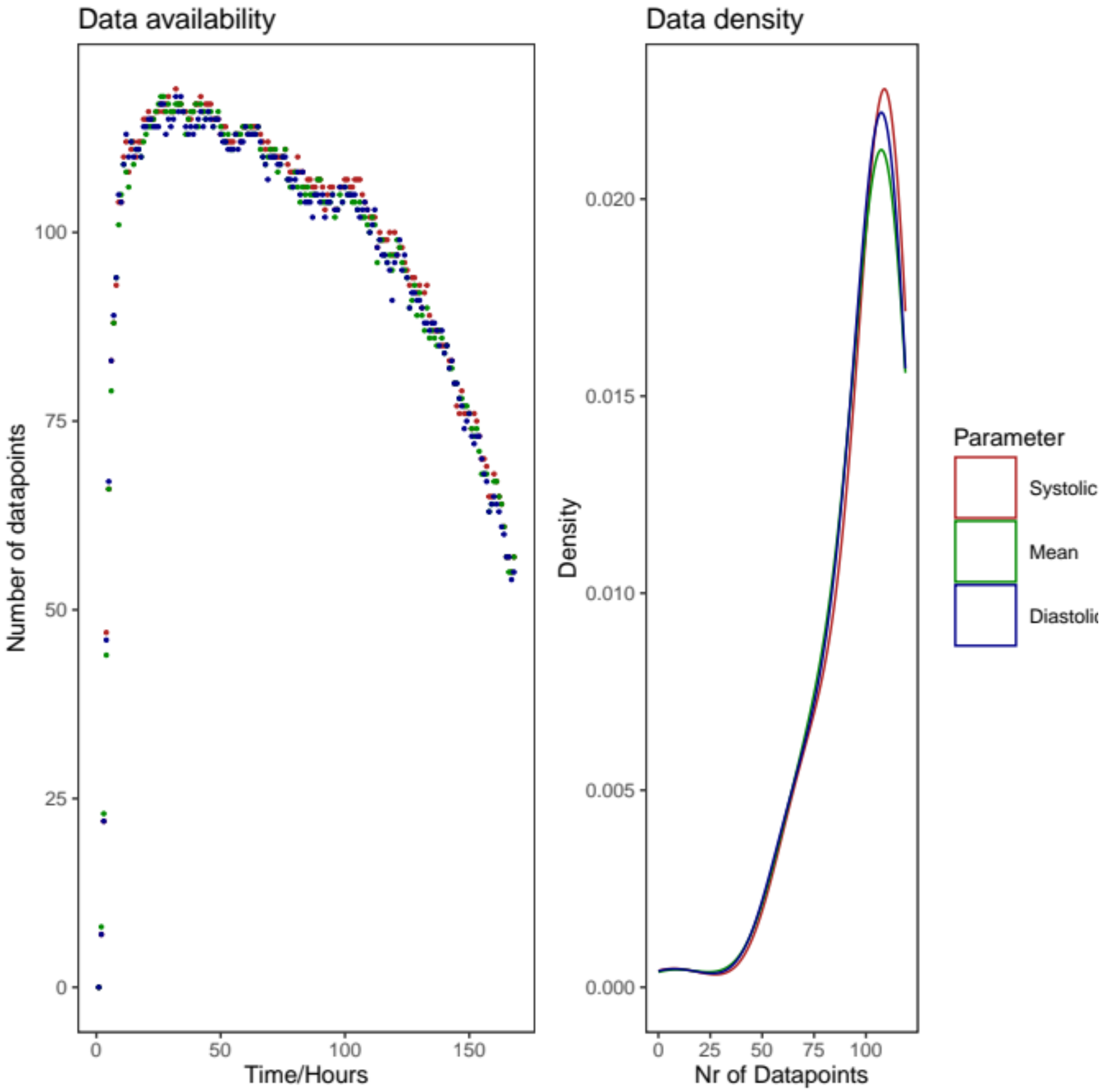

Supplementary Figure 2. Data availability per patient after outlier removal for the first 168 hours. Red indicates systolic data, green mean data, and blue diastolic data (left). Data density for the dataset,

w Only

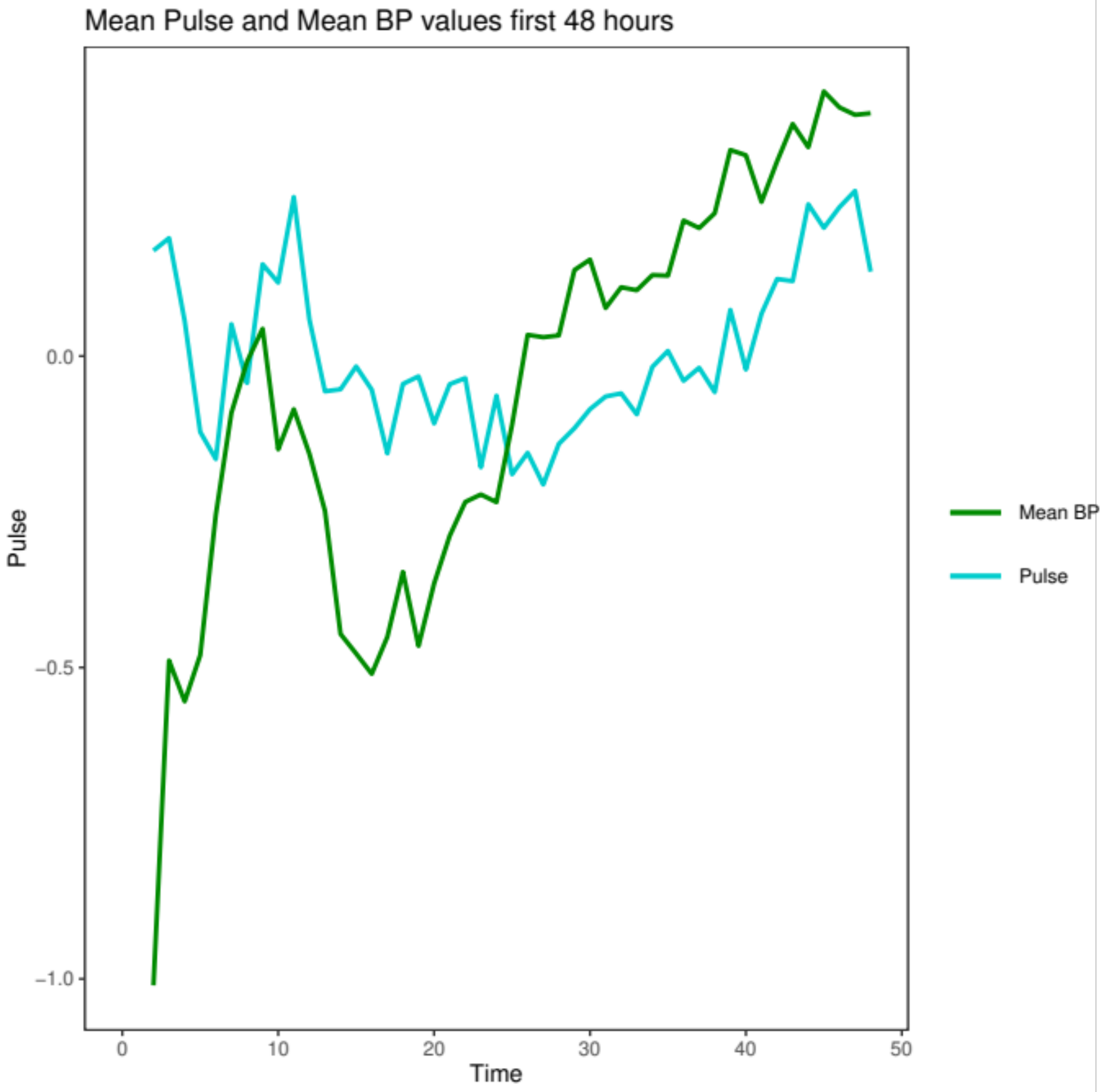

Supplementary Figure 3. Mean blood pressure and pulse rate for the first 48 hours, data has been scaled and centered to enable comparison of the respective dynamics.

Only

Tables

Supplementary Table 1. Granger test statistics for comparison of various conditions. Significant test statistics (in both directions) in bold. Tests were performed with frequency=24 (i.e., one day) and lag=6.

Test	Parameter	F 1vs2	P-value 1vs2	F 2vs1	P-value 2vs1
Expired Vs Survived	S	0.7114	0.6411	1.7362	0.1181
	M	1.0648	0.3874	1.2985	0.2628
	D	1.0166	0.4177	2.7913	**0.01401 ***
IVH vs NoIVH	S	0.672	0.6725	1.3972	0.221
	M	1.8902	0.08768 .	0.7394	0.6189
	D	1.8553	0.09386 .	0.838	0.5429
NEC Vs NoNEC	*S*	1.0188	0.4163	2.2885	0.03959 *
	**M**	2.1973	**0.04762 ***	1.4941	0.1856
	D	2.2921	**0.0393 ***	4.1012	**0.0008705 *****
NEC And IVH	S	1.6417	0.1412	1.4911	0.1866
	M	1.1992	0.3112	1.1367	0.3451
	**D**	3.7469	**0.001853 ****	2.7138	**0.01647 ***
Week22 Vs Week 23	S	2.1184	0.05588 .	0.3234	0.9237
	M	3.7312	**0.001924 ****	1.7925	0.1061
	D	1.0904	0.372	2.2688	**0.04126 ***
Week22 Vs Week 24	S	1.598	0.1533	**1.4297**	0.2086
	M	0.6603	0.6818	1.1013	0.3656
	D	1.2781	0.2723	0.8565	0.5291
Week23Vs Week 24	S	6.5905	**4.651e-06 *****	**1.7821**	0.1081
	M	2.6125	**0.02034 ***	2.13	0.05452 .
	D	4.5404	**0.0003415 *****	2.0958	0.05839 .
Csec Vs Natural	S	3.9531	**0.001194 ****	2.2704	**0.04107 ***
	M	2.6132	**0.02031 ***	1.765	0.1117
	D	1.5071	0.1813	2.1612	0.05121 .
ChorioA Vs NoChorioA	S	3.2583	0.005236 **	1.0051	0.4252
	M	0.8812	0.5109	1.8826	0.08899 .
	D	2.337	0.03586 *	1.1103	0.3602

Confidential: For Review Only

Supplementary Table 2. Output summary of generalized linear model for conditions and analyzed numerical variables. Model: IVH Grade ~ Week + Inotrope/vasoactive drug + NEC + >46mmHg at any time + %Time spent at>46mmHg + Nr.Fluctuations. Data were scaled and centered prior to analysis.

Condition	Estimate	Std. Error	z value	Pr(>|z|)	Sig Level
Week	-0.19562	0.09005	-2.172	0.029833	*
NECTRUE	-0.21959	0.28044	-0.783	0.433605	
GT46mmHgTRUE	0.59868	0.26861	2.229	0.025825	*
PercGT46mmHg	-0.18566	0.1585	-1.171	0.24146	
NrFluc168	-0.34582	0.09755	-3.545	0.000393	***

Supplementary Table 3. Outcome of Fisher's exact tests for patient outcome. Significant test statistics in bold, weakly significant test statistics in bold italics.

Test of proportions: Condition	Fisher's exact p-value	Odds Ratio	Sig. Diff.
Expired patients with IVH diagnosis Vs. No diagnosis	***0.0616***	***0.428422***	No
Expired patients with NEC diagnosis Vs. No diagnosis	**0.02968**	**0.3014454**	Yes
Expired patients with IVH&NEC diagnosis Vs. No diagnosis	**0.01159**	**6.036246**	Yes
IVH Diagnosis GA week 22 vs week 23	0.1812	0.2950553	No
IVH Diagnosis GA week 22 vs week 24	0.4857	0.4885168	No
IVH Diagnosis GA week 23 vs week 24	0.2462	1.656861	No
Patient Exceeded/Did not exceed 46mmHg and Expired	0.2567	1.735024	No
Patient Exceeded/Did not exceed 46mmHg and diagnosed with IVH	0.1698	1.833681	No
IVH Diagnosis Csec vs Norm	0.3632	0.6829341	No
IVH Diagnosis ChoriaA diagnosis vs No diagnosis	0.06121	0.4342773	No

Description of data extraction and statistical analyses

Data extraction, normalization, and removal of outliers

The output from the data monitoring systems in Microsoft Excel format were converted to TSV files and an in-house developed perl-script was used to average the values (recorded on a nominal one datapoint per minute but displaying gaps in the data) on a per-hour basis in order to compensate for gaps in the data. Furthermore, time elapsed since birth to beginning of monitoring was taken into account, resulting in normalized data comparable between patients.

Due to rapidly decreasing amount of data after the 168-hour mark (corresponding to approximately one week after birth), a cut-of at this hour was implemented and no later data-points were included in the analysis.

In order to account for spurious data, caused by measurement errors and other factors unlikely to have a biological/physiological basis, interquartile range criterion (IQR) was used to clean the data and remove outliers. This resulted in removal of 299, 602 and 535 datapoints for the systolic, mean and diastolic datasets, respectively.

Confidential: For Review Only

Granger's causality tests for time-series data

Granger causality test is a statistical method used to determine whether one time series is a useful predictor of another time series. In other words, it can determine if a change in one variable can be used to predict the change in another variable. In the analysis presented here, it was used to determine if the BP curves were statistically similar enough that one curve could be used to predict another for a given set of conditions, i.e., if statistical similarity exist between BP development (e.g., inotrope/vasoactive drug having been given or not). If one curve is not able to predict the development of another, we can conclude that they are statistically significantly different, given that this is significant in both directions.

To account for the fact that the data was analysed in aggregate, i.e., the mean of all patients exhibiting a specific set of conditions, and that patients are likely to display variation in individual development, a frequency of 24 hours and a lag of 6 hours was used in the comparisons.

The "lmtest (version 0.9-40)" R package was utilized for all time-series analysis.

Effect of BP in specific "bands," and identification of dangerous BP thresholds

The procedure described in Vesoulis *et al.* was repeated for the time each patient spent in specific "bands," corresponding to percent of total measurement time spent between specific defined thresholds of blood pressure.

However, no correlation between outcome (e.g., grade of IVH, patient expired) and percentage spent at specific BP values could be found in our data. However, a statistically significant difference was detected regarding if the patient at any time had exceeded the threshold identified by Vesoulis *et al.,* corresponding to 46mmHg for mean blood pressure.

Further, an attempt was made at identifying alternative thresholds with greater statistical effect than the threshold identified by Vesoulis *et al.* by receiver operating characteristic (ROC) analysis, but no threshold value with greater power could be detected.

Analysis of number of fluctuations in blood pressure, i.e., stableness

To analyse the number of fluctuations/how stable the blood pressure was for each patient, an analysis was performed where all patients with a maximum of half the time-points missing, and the missing time-points fixed by linear interpolation with the R na.interp function were included in the analysis. Subsequently, the number of times each patient blood pressure crossed its own 24-hour rolling mean was counted. This concept is illustrated for a singular patient in supplementary Figure 4, where the red line corresponds to the rolling mean and the black line corresponds to the actual BP values.

Confidential: For Review Only

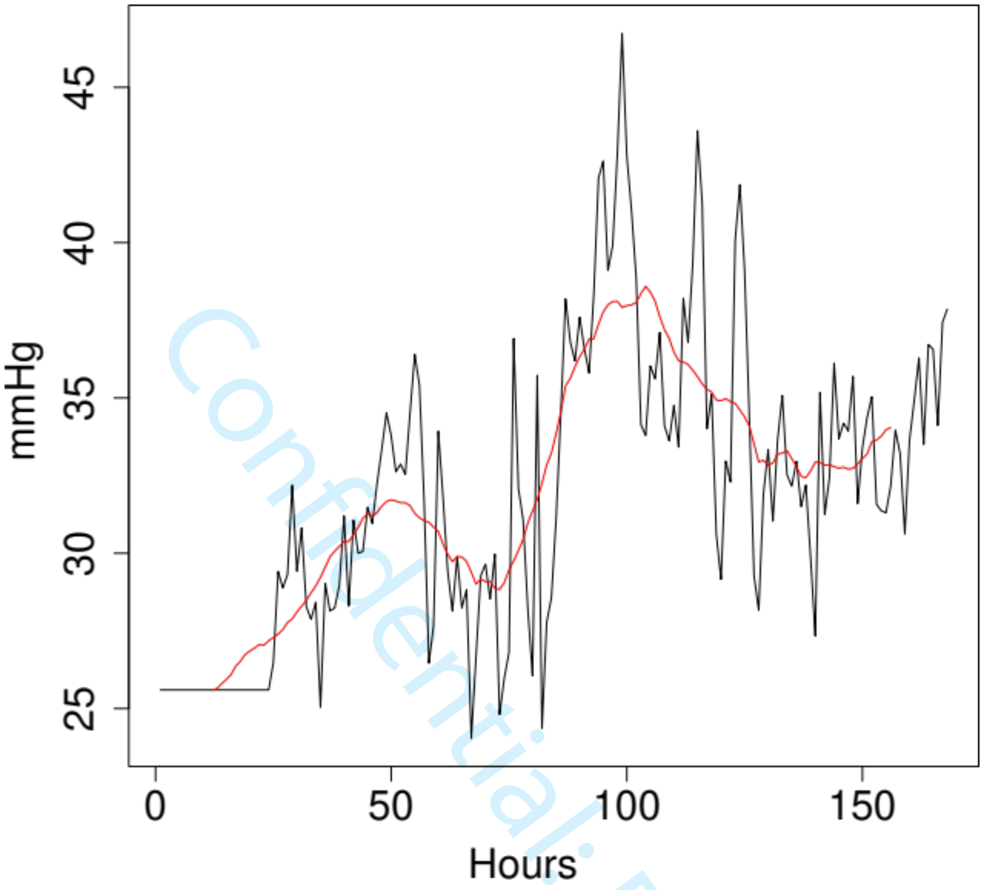

Supplementary Figure 4. Illustration of concept of analysis of number of fluctuations.

In other words, a numerical value was obtained for each patient measuring how many fluctuations took place around the rolling mean, giving a measurement indicating the how stable blood pressure is/slow response to divergent blood pressure.

Determination of nadir-point for individual patients

To detect the nadir point for individual patients, data-series with a maximum of 25% of the data-points missing were selected, and the R function cpt.mean from the "changepoint (version 2.2.4)" R package was used after linear interpolation with the R na.interp function to fix missing datapoints. In order to avoid spurious detections, the analysis was limited to the period between 8 and 36 hours. The parameters for cpt.mean were set as follows: penalty="MBIC",pen.value=0,method="AMOC",Q=5,test.stat="Normal",class=FALSE, param.estimates=TRUE,minseglen=1.

Evaluation of significant factors for inclusion in modelling

In order to evaluate potential factors to include in the general linearized model, a number of different models were evaluated, leading to the final model incorporating factors either previously described as being significant in the literature, or where a Fisher's exact test for our own data indicated that the outcome was numerically different, or where other analysis had suggested that explanatory power was present as in the case of number of fluctuations. A presentation of the factors evaluated can be found in Supplementary table 3. Akaike Information Criterion (AIC) was used to select the final model providing the best fit. The final model corresponded to: IVH Grade ~ Week + Inotrope/vasoactive drug + NEC + >46mmHg at any time + %Time spent at>46mmHg + Nr.Fluctuations.

Confidential: For Review Only
